# High concentrations of plastic hidden beneath the surface of the Atlantic Ocean

**DOI:** 10.1038/s41467-020-17932-9

**Published:** 2020-08-18

**Authors:** Katsiaryna Pabortsava, Richard S. Lampitt

**Affiliations:** National Oceanography Centre, European Way, Southampton, SO14 3ZH UK

**Keywords:** Environmental sciences, Ocean sciences, Marine chemistry

## Abstract

Concern over plastic pollution of the marine environment is severe. The mass-imbalance between the plastic litter supplied to and observed in the ocean currently suggests a missing sink. However, here we show that the ocean interior conceals high loads of small-sized plastic debris which can balance and even exceed the estimated plastic inputs into the ocean since 1950. The combined mass of just the three most-littered plastics (polyethylene, polypropylene, and polystyrene) of 32–651 µm size-class suspended in the top 200 m of the Atlantic Ocean is 11.6–21.1 Million Tonnes. Considering that plastics of other sizes and polymer types will be found in the deeper ocean and in the sediments, our results indicate that both inputs and stocks of ocean plastics are much higher than determined previously. It is thus critical to assess these terms across all size categories and polymer groups to determine the fate and danger of plastic contamination.

## Abstract (web summary)

The risks posed by plastic contamination of the ocean cannot be assessed as their amount and location remain largely unknown. Here the authors show that large quantities of microplastics exist below the ocean surface over the entire Atlantic in quantities greater than previously estimated.

## Introduction

Marine microplastics (10–1000 µm) belong to the continuum of the discarded plastic debris that enters the ocean from land-based and marine sources^1^. The pathways of plastic input are very diverse and include riverine^2^ and atmospheric transport^3^ from coastal and inland areas, illegal dumping activities, erosion of legacy refuse dumps, and direct at-sea littering from shipping, fishing and aquaculture activities^1,4^. The ubiquitous presence of microplastics in the marine environment raises concerns over damage they could cause to oceanic ecosystems and eventually to human health^5,6^. Yet, the scientific evidence for the present and future risks from microplastics is far from robust as sources, exposure levels and harm from these contaminants are all poorly constrained. Although a significant body of data on (micro)-plastic loads in the ocean has been collected, the geographical spread of the measurements is sparse and have been mostly focussed on plastic particles greater than 250 µm in size from the surface waters and seafloor (ref. ^7^ and references therein). Abundance and distribution of microplastics, especially those in smaller size categories (<250 µm) throughout the vast ocean interior remain virtually unknown except for one full-depth (8–4400 m) survey in the Arctic Central Basin^8^. This leaves a significant knowledge gap, as the presence of microplastics >11 µm in the deep-sea sediment^9^ indicate that removal from the surface ocean to the abyss does take place. Given the current lack of knowledge about the location and fate of microplastics in most of the ocean volume, the loads of oceanic plastics floating in the surface ocean cannot be balanced by their mass fluxes from land and marine sources^7,10^. The estimated inputs of plastic debris into the ocean^2,4,11^, in turn, are also massively uncertain and require robust empirical assessments on a global scale.

The additional challenge/uncertainty comes from the versatility of plastic materials and hence the necessity to assess the pollution with classes/polymer types of microplastics rather than considering them as a single material^2^. The extremely wide range of physical and chemical properties of different plastic types would in part determine the extent and rate of their transformations (e.g. fragmentation^12,13^, degradation^14^, aggregation^15^) and interactions (biofouling^16^ and ingestion^17,18^) in the ocean and thus their persistence and impacts on the biota therein.

**Fig. 1:**
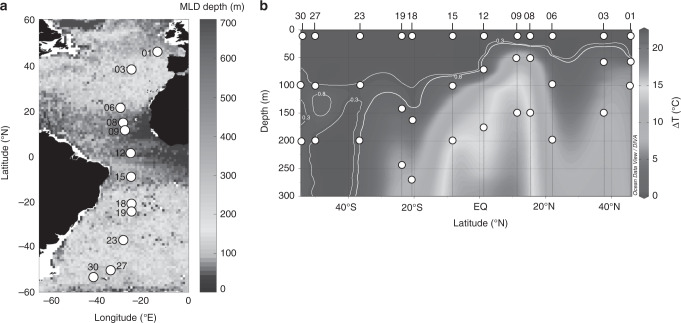
Sampling locations for microplastics. **a** Sampling sites (numbered white circles) superimposed on a climatology of maximum monthly mixed layer depth (MLD, m) in the Atlantic Ocean compiled using Argo profiles^79,80^**b** Latitudinal section showing sampling locations (numbered) for microplastics (white circles) in the water column in relation to the depth of mixed layer, defined as an absolute change in temperature (Δ*T* (°C)) with respect to an approximately uniform region of temperature at 10 m below the ocean surface^25^. Vertical profiles of temperature were collected at each station prior to the deployment of SAPs (ref. ^23^). Isotherms (white contours) of Δ*T* = 0.8 °C marked the base of the mixed layer at stations 01 to 19. Due to intense mixing at stations 23, 27, and 30, the fixed temperature criterion was lowered to Δ*T* = 0.3 °C (ref. ^25^).

Here, we assessed the pollution from polyethylene (PE), polypropylene (PP) and polystyrene (PS) litter at 12 locations on a 10,000 km North–South transect of the Atlantic Ocean (Fig. 1). The polymer groups investigated are the most common commodity plastics that are mainly used for packaging. They thus have a short lifetime and a high contribution to the content of the global plastic waste (56%)^19^. Recent meta-analysis^20^ also identified PE, PP and PS as the most abundant polymers in the marine environment, although their distribution in the open ocean and especially its interior was poorly constrained. We measured penetration of PE, PP and PS particles down to 25 µm in size from the near surface to the ocean interior below the maximum depth of upper ocean mixing (>200 m). We discuss our findings in the context of previous observations and estimates of plastic pollution in the Atlantic, both horizontally and with depth. We provide a basin-scale assessment of the magnitude of pollution by these polymers in the upper 200 m and relate these data to the previously calculated plastic inputs to the ocean over the past 65 years.

## Results

### Field observations

To quantify and characterise the horizontal and vertical abundance of PE, PP and PS, at each station of the transect we collected suspended marine particles including plastics using in situ stand-alone pumps (SAPs; Challenger Oceanic Ltd.)^21,22^ deployed simultaneously at three discrete depths (Fig. 1b and Supplementary Table 1; ‘Methods’). The shallowest sampling depth was always at 10 m below the surface to obtain concentrations of microplastics representative of the upper water column (Fig. 1b). We collected microplastics at two depths below the base of mixed layer to measure their dispersal into the ocean interior. The mixed layer depth (MLD) was determined from the conductivity–temperature–depth profiles collected prior to each deployment of the pumps (refs. ^23,24^) and employing a fixed temperature-based criterion (Δ*T* = 0.8 °C for stations 01–19 and Δ*T* = 0.3 °C for stations 23, 27 and 30; Fig. 1b; see ref. ^25^). On our latitudinal transect, the MLD was between 28 and 140 m. The intermediate sampling depth was selected to be at ~10–30 m below the MLD (Fig. 1b; Supplementary Table 1). The deepest (mesopelagic) layer of particle collection was 100 m below this intermediate sampling horizon, a depth well into the interior of the ocean (Fig. 1b; Supplementary Table 1) and isolated from the ocean surface for decades^26^.

All steps involving sample collection, processing and analysis were performed in the air-controlled environment and using clean, pre-combusted and where possible non-plastic laboratory-ware (‘Methods’). Particle collection with SAPs offered significant advantages with respect to the volumes of seawater filtered (507–1534 L per SAP; Supplementary Data 1) and prevention of air-borne contamination (‘Methods’). No PE, PP and PS microplastics were detected in all procedural blanks (Supplementary Figs. 1 and 2) indicating contamination-free sampling and analysis (also see Supplementary Methods). Following the removal of particulate organic material with KOH^27,28^, microplastics were pre-concentrated on a stainless-steel mesh with 25 µm aperture to be detected and characterised (polymer type and size) using Fourier-Transform infrared (FTIR) imaging at 25 µm resolution (‘Methods’).

We report the concentrations of polymer-specific microplastics as particle number per unit volume (particles m^−3^) for a comparison with previous studies. Imaging IR provides two-dimensional (2D) properties (length, width, area) of individual particles, which, along with the respective particle count data, was used to estimate polymer-specific mass concentrations (µg m^−3^: see ‘Methods’, ref. ^24^ and Supplementary Data 1) and subsequently their respective loads in the ocean (‘Methods’). We calculated particulate mass using the same procedure and assumptions of particle shape, thickness and density as in previous studies^10,29–32^ but report the lowest values to provide the most conservative estimates of mass concentrations (detailed in Supplementary Methods and Supplementary Fig. 3). Mass of individual microplastics <300 µm have not been measured directly in bulk marine particle samples. We present empirical information about the size and mass of polymer-specific plastic penetrating deeper into the ocean interior, crucial for understanding and predicting the global inventory of marine plastic debris and their sources.

### Abundance and distribution in the Atlantic Ocean

**Fig. 2:**
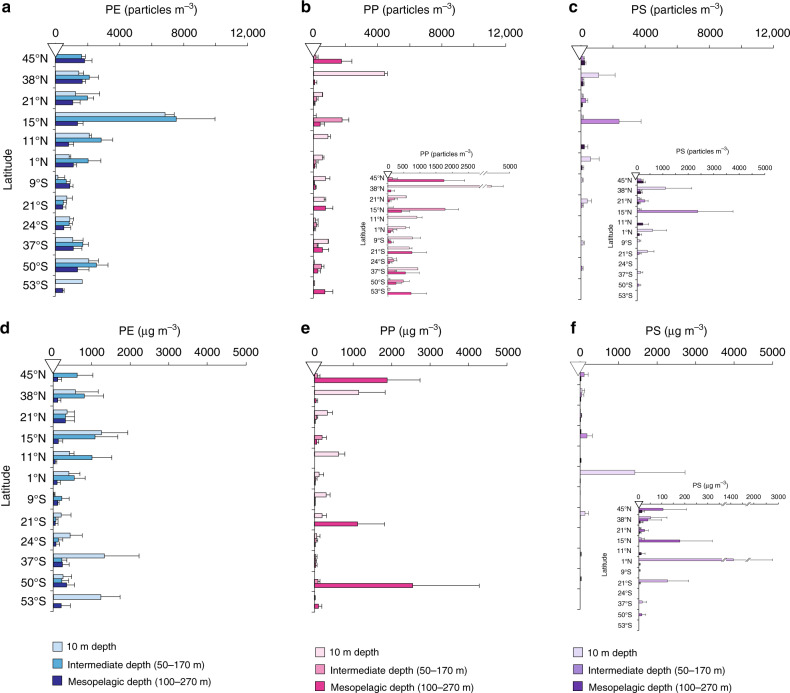
Microplastics abundance in the study area. **a**–**c** particle number concentrations (particles m^−3^) and **d**, **e** mass concentrations (µg m^−3^) of the polymer groups at each station and depth layer. No near surface (10 m) sample was taken at 45°N (marked with white triangle) due to pump failure. In **a**–**c**, the height of the bar shows the particle number concentration derived from the mean microplastic count in four imaged areas of a sample^24^ and scaled to the known total area filtered and the known sample volume of the investigated sample fraction (see ‘Methods’ and Supplementary Data 1). In **d**–**f**, the height of the bar shows the mass concentration of microplastic derived from particle number concentration data and two-dimensional properties of detected individual particles using Method IV (ref. ^10^; see Supplementary Methods and Supplementary Data 1). All error bars are one standard deviation showing uncertainty propagated through the calculations. Only upper error bar (+1 s.d.) is shown for clarity. Note a magnified scale for concentrations of polypropylene (**b**) and polystyrene (**c**, **f**).

PE, PP and PS microplastics were found at all stations in number and mass concentrations that varied by several orders of magnitude horizontally and with depth (Fig. 2). Overall, PE was the most abundant and pervasive polymer group with significantly higher number and mass concentrations (mean ± s.d., 1602 ± 1551 particles m^−3^ and 389 ± 377 µg m^−3^) compared to PP (490 ± 822 particles m^−3^ and 262 ± 568 µg m^−3^) and PS (180 ± 439 particles m^−3^ and 58 ± 241 µg m^−3^) (for all, Mann–Whitney *U* test: *p* < 0.001, *α* = 0.01). PE microplastics were identified in all samples except for the intermediate depth layer of the southernmost station (53°S). Note that no PP or PS microplastics were found at this sampling location, although other types of polymers such as polyamide and cellophane were present (Supplementary Data 2), from which we conclude that the absence of PP and PS was not a consequence of faulty sampling. The presence of PP and especially of PS below the MLD was patchy. Only 67% of the samples from the intermediate depth layer contained PP while PS microplastics were encountered in 60% of the surface samples and in <50% of the deeper ones.

**Fig. 3:**
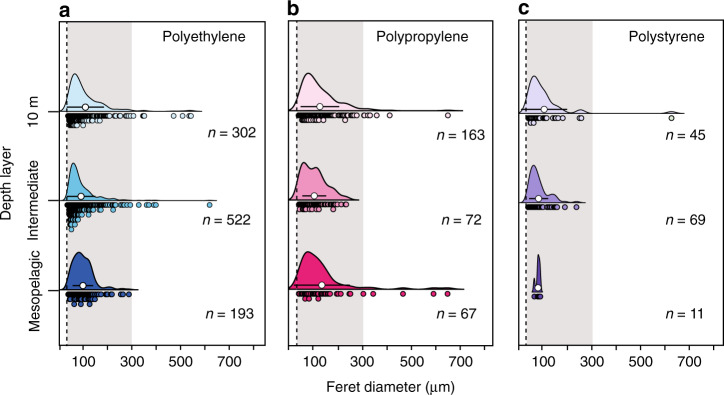
Size distribution of microplastics with depth. **a** Polyethylene, **b** polypropylene and **c** polystyrene. Data points making up the distributions are shown as circles of the respective colour. The width of each distribution was scaled to be equal to allow the inter-comparison of the distributions regardless of the number of data points (*n*). The white circles indicate mean particle sizes with horizontal bars as ±standard deviation. Dashed line marks the lowest particle size measured (32 µm) in this study. The dominance of microplastics <300 µm in size is highlighted in grey. Source data set is provided in ref. ^24^.

The size of all the detected PE, PP and PS microplastics was measured as maximum diameter (Feret diameter) and ranged from 32 to 651 µm (mean = 81 µm, *n* = 1444; Fig. 3). The majority of the polymer-specific microplastics were <100 µm (PE = 68%, PP = 49% and PS = 67%) with the peak size distribution observed in the range 50–75 µm for all the polymer groups (Fig. 3). Only a small fraction of all three polymer groups were >300 µm: for PE, the contribution of this size fraction was 1.1% numerically, while the respective values for PP and PS were 2.5% and 0.7%, respectively. The PP microplastics had a higher contribution of 100–200 µm particles (40%) compared to PE (27%) and PS (18%), which explains overall significantly larger average sizes (±s.d.) of PP microplastics (117 ± 76 µm; *n* = 302) than those of PE (96 ± 61 µm; *n* = 1017; Mann–Whitney *U* test: *W* = 147,085, *p* = 1.42 × 10^−9^, *α* = 0.01) and PS (87 ± 64 µm; *n* = 125; Mann–Whitney *U* test: *W* = 29,341, *p* = 8.94 × 10^−8^, *α* = 0.01).

**Fig. 4:**
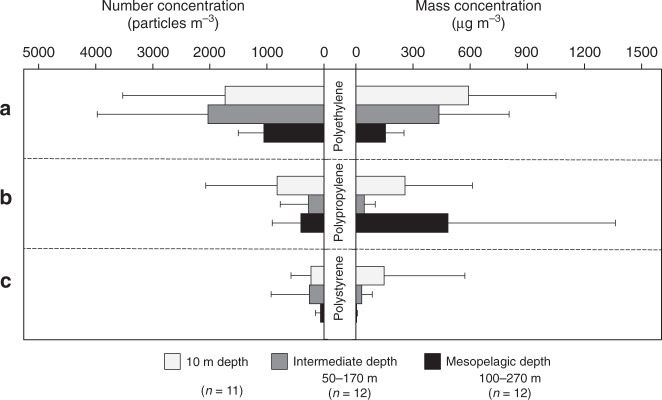
Vertical distribution of microplastics in the study area. The bars show particle number (particles m^−3^) and mass concentrations (µg m^−3^) of **a** polyethylene, **b** polypropylene and **c** polystyrene averaged over all sampling stations at each depth horizon. Data presented as mean values with upper error bars (+1 s.d.) for clarity. In figure legend, letter *n* in parentheses indicates the number of stations sampled at the respective depth horizon (Supplementary Table 1 and Supplementary Data 1).

We find elevated number and mass concentrations (mean ± s.d.) of PE (1732 ± 1793 particles m^−3^ and 591 ± 460 µg m^−3^), PP (822 ± 1250 particles m^−3^ and 258 ± 354 µg m^−3^) and PS (228 ± 350 particles m^−3^ and 148 ± 424 µg m^−3^) in the near-surface waters, although considerable quantities of these microplastics were found below the MLD (Fig. 2, Fig. 4). As such, by count, mean PE and PS abundances were comparable in the surface and right below the MLD (Fig. 4a, c). In the mesopelagic, the average abundance decreased by a factor of 2 for PE (1052 ± 452 particles m^−3^) and dropped ~4-fold for PS (mean, 62 ± 90 particles m^−3^). By mass, however, PE and PS concentrations declined steadily with depth (Fig. 4a, c). The mass loss with depth was faster for PS microplastics than for PE. The apparent mismatch between the vertical patterns of number and mass concentrations of PE and PS was in part attributed to a significant decrease in particle size between the surface and intermediate depth layers (Fig. 3a, c). Here the mean (±s.d.) size of PE and PS microplastics changed from 105 ± 74 to 89 ± 57 µm (Mann–Whitney *U* test: *W* = 101,077, *p* = 0.000582, *α* = 0.01) and from 102 ± 95 to 78 ± 38 µm (Mann–Whitney *U* test: *W* = 249.5, *p* = 0.01077, *α* = 0.01), respectively. Contrary to PE and PS, the mean number and mass concentrations of PP were the lowest at the intermediate depth layer (mean ± s.d., 271 ± 496 particles m^−3^ and 44 ± 58 µg m^−3^; Fig. 4b). PP microplastics were also smaller in size at this depth layer (mean ± s.d., 102 ± 47 µm) than the particles captured in the surface (mean ± s.d., 119 ± 66 µm) and mesopelagic waters (mean ± s.d., 132 ± 112 µm) (Fig. 3b), although these differences were not significant based on Mann–Whitney *U* statistics.

## Discussion

This study provides the wide-scale depth-resolved data on pollution of the Atlantic Ocean by PE, PP and PS microplastics. Our observations reveal very high concentrations of these polymers in the size range 32–651 µm spreading across all latitudes and penetrating from the near surface ocean, through the mixed layer and into the ocean interior (>200 m).

Overall, the relative mass concentrations of the polymer-specific microplastics in our samples (PE > PP > PS) was consistent with the polymer composition of plastic waste generated globally^19^ and captured in the surface ocean and at the seabed (review by ref. ^20^ and references therein).

A direct comparison of our near-surface abundance data with the previous Atlantic studies of microplastic pollution is challenging as nearly all of them applied different sampling and analytical approaches. As such, our study assessed in a consistent, targeted manner the smaller size category of microplastics across an extensive ocean region and at three discrete depths and analysed them using FTIR imaging technique. Our method enabled the detection of very small particles and did not require visual selection of suspect plastics prior to analysis^33^. We thus detected much higher near-surface abundances of the examined polymer groups (range combined, 990–6999 particles m^−3^) compared to the Atlantic records of pre-selected, bulk microplastic debris in size range ≥10 µm (range, 13–801 particles m^−3^; ref. ^34^). Similarly, we found more PE (64 particles m^−3^), PP (29 particles m^−3^) and PS (12 particles m^−3^) debris in size range >250 µm than the earlier surveys of bulk microplastics in the Atlantic (e.g. range 0–8.5 particles m^−3^ in ref. ^35^ and 0–22.5 particles m^−3^ in ref. ^36^). Our polymer-specific concentrations were, however, significantly lower than the load of bulk plastic debris of >25 µm in size detected in the Arctic Sea Ice (10^5^–10^8^ particles m^−3^) using the same detection approach^33^. Note that PE also dominated the polymer composition in most of the Arctic ice cores examined in ref. ^33^.

Despite the different methodologies, our near-surface measurements are comparable to the wind-corrected estimates of 25–1000 µm bulk microplastics floating in the North Atlantic (610–36,000 particles m^−3^; ref. ^31^). The particle size distribution of PE, PP and PS microplastics was dominated by 50–80 µm fraction, consistent with findings of ref. ^34^. The combined mass concentration of PE, PP and PS in the near surface (range, 259–1969 µg m^−3^) was also of the same order of magnitude as the concentrations of the floating bulk plastic debris of >300 µm in size (100–1000 µg m^−3^ and ref. ^10^ and 5–14,000 µg m^−3^ in ref. ^31^). Further to refs. ^31,34^, our findings now provide a strong support for the smaller-sized microplastics being a dominant constituent of the oceanic plastic inventory, previously unaccounted by the common sampling techniques and hence not included in the estimates of the oceanic burden of plastics^7,37,38^.

The observations of microplastic abundance in the open ocean setting and at depths below the mixed layer were only made in the Arctic Central Basin^8^. These data are also limited to pre-selected microplastics of >250 µm size class, of which 96% were synthetic fibres. The remaining fraction were non-fibre microplastics of polymer types other than PE, PP and PS and combined concentrations 0–4 particles m^−3^, which hinders the direct comparison with our data.

With respect to the basin-wide distribution pattern, we observed no prominent increase in subsurface abundance of small microplastics in the Atlantic subtropical gyres, where larger floating plastics, pre-cursors of microplastics, seem to accumulate according to the previous sampling efforts^10,34,39,40^ and predictions from the large-scale surface ocean transport models^7,37,38^. In the northern gyre, the elevated abundance of PE, PP and PS microplastics were only seen at 15°N (Fig. 2) and were likely advected from Cape Verde (located ~600 km east of the sampling site (Fig. 1)) by prevailing currents and winds. Concentrations of all three polymer groups were the lowest at stations 15–19 in the South Atlantic subtropical gyre (Fig. 2). At present, this spatial mismatch cannot be explained with certainty. One reason is that the full extent of accumulation of different size classes of plastic debris in the gyres has not yet been measured, while most of the ocean surface and subsurface waters is also undersampled^7^. This introduces serious uncertainties in identifying distribution patterns of plastic contamination on basin and global scales^7,41^. Noteworthy is the fact that earlier surveys^10,34,39,40^ report highly variable abundance of net-collected plastics across the gyres with different locations of the measured hotspots in the Atlantic relative to our sampling sites. Another reason concerns the fundamental processes that supply, distribute, transform and remove plastics in the ocean which need to be constrained to explain the variations in plastic abundance in the sea surface and at depth. As such, a surface inventory of plastics on any size class at a given location reflects inputs and removal rates over time. The input rate is a function of the amount of plastic debris entering the ocean and the rate at which they fragment to sizes that can be captured by the available sampling techniques. Once in the surface ocean, plastics are distributed around by the prevailing winds, surface currents and small circulation features^7,42,43^. Removal processes will dilute plastic concentrations in the surface and determine their abundance in the ocean interior^34^. These processes include advection^37,44^, ingestion by zooplankton^17^ and larger marine organisms^45^, gravitational sinking following biofouling and incorporation of microplastics into phytodetrital aggregates^15^ and faecal material^18^. The mechanistic nature, strength and rates of these redistribution and removal processes are currently unknown but will likely vary within different regions of oceanography in the Atlantic and globally. Our depth-resolved assessment of microplastic contamination over spatially extensive transect in the Atlantic reveals the complexity of the interactions between oceanic processes and plastic debris. An intensive, coherent and repeated sampling effort, similar to the annually repeated AMT voyages, or involving autonomous in situ observations, are required to resolve these interactions in different oceanic regions. This is critical if we are to understand and predict the fate and impacts of plastics debris on the marine ecosystems.

Our study demonstrates a strong heterogeneity in horizontal and vertical abundance of the individual plastic types. The ubiquitous presence of PE and PP microplastics in the surface ocean^33–35^ and sediments^9^ has been documented previously. We now show that the sub-surface contamination with these polymers is also pervasive and reaches very remote areas of the South Atlantic (Fig. 2a, b). PE and PP are the most used and littered globally^19^ and are also initially buoyant in seawater and thus could travel over long distances before being lost into the ocean^33^. It is possible that the elevated quantities of PE and PP microplastics (up to 2553 and 726 particles m^−3^, respectively) found around South Georgia (stations 27 and 30) were advected by the Southern ACC Front travelling from the Rapa Nui garbage patch in the South Atlantic^39,46^ or by the Sub-Antarctic Front, which could have entrained plastic-contaminated waters carried by the Brazil Current^39^. Input from local fishing activities around the archipelago is also likely. For example, parts of fishing gear such as braided ropes of longlines and trawls are often made of PE and PP or composites of PE with other polymers^47–49^.

Of the examined groups, PS was the only polymer to show a significant southward decrease in its surface mass abundance (Mann–Whitney, *W* = 233, *p* = 0.0053, *α* = 0.01; Fig. 2f), which is likely due to overall lower production, consumption and waste of this polymer by the countries in the Southern Hemisphere^4,50^. PS microplastics were scarce in the near surface, consistent with previous observations^35,40^; they were rarely encountered in the mesopelagic layer and not detected at this depth in the South Atlantic (Fig. 2c, f). Relatively low stability and high degradation rates of PS in seawater could be one explanation for the low abundance as well as smaller particle sizes of this polymer compared to that of PE and PP^51^. In addition, PS is produced in two distinct chemical grades, which could impact its fate in the water column. Microplastics of crystal-grade PS (used in houseware and rigid packaging) are denser than seawater (*ρ* = 1.04–1.05 g cm^−3^), and thus might be more prone to transport down the water column and surpass the depths sampled in our study. Alternatively, but not exclusive, microplastics derived from the foam expanded PS material, which is 98% gas by composition with density of ~0.05 g cm^−3^, might be preferentially retained above the shallowest depth sampled in our study and/or dispersed horizontally by prevailing surface currents^44^ and winds.

We note a higher error of PS concentrations compared to that of PE and PP (Fig. 2) due to low absolute counts of PS microplastics in the individual samples (Supplementary Table 1). The importance of class-based assessment of microplastics in environmental samples was suggested previously^2^. Our data now demonstrate the importance of tailoring the methods of microplastics sampling and analysis to specific polymer types with a consideration of their likely abundance in the environment. As such, targeted extraction of polymer types of interest, pre-concentrating them by filtering larger volumes of water and scanning larger image areas on the filter could reduce the uncertainty when studying relatively rare plastics, such as PS. Similar approach has been developed for measurements of major and trace elements in the ocean^52^, which could be used as a guideline for improving and harmonising methods in marine plastic research.

We report the combined mass concentrations of PE, PP and PS microplastics below the MLD and in the mesopelagic to be on average (±s.d.) 511 ± 440 and 642 ± 916 µg m^−3^, respectively. Although extremely variable with latitude, these values are of the same order of magnitude as the mass loads of larger plastic litter, pre-cursors of microplastics, reported to float in the Atlantic^10,31^. The observed conservation of plastic mass at depth points to downward transport of surface plastics after fragmentation. It also demonstrates that the removal of these small microplastics into the ocean interior is an important sink that prevents plastic accumulation in the surface waters.

The predominance of microplastics <100 µm at all our sampled locations indicates that the horizontal dispersal of microplastics and their loss into the ocean interior is a size-selective process^10,34,42^. We hypothesise that some portion of the PE, PP and PS debris was released into the marine environment in sub-millimetre size (e.g. as deliberately manufactured microplastics), while mechanical forces and photochemical processes fragmented larger plastics into microplastics both in situ and during their transit to the remote Atlantic waters. Regardless of the provenance, the small size of microplastics appears to be an important trigger for downward transport to occur. Smaller microplastics are more prone to vertical dispersal by mixing and diffusion, especially within the mixed layer^30,34,43^. Biofouling of plastic surfaces, a process that reduces buoyancy of plastics sufficiently to cause them to sink^53–55^, has been shown to be faster for smaller particles due to their high surface-to-volume ratio^56^. In turn, biofilm-covered microplastics aggregate more rapidly with marine snow^57^, which could facilitate their downward export to the deep ocean. A preferential ingestion of smaller plastics by marine zooplankton has also been observed^17,58^, with implications for their subsequent incorporation into fast-sinking faecal pellets^18^.

The overall absence of a clear pattern in vertical abundance of the examined polymer groups indicates that their supply, distribution and fate, and hence, residence time in the water column, are affected by diverse and complex processes^10^. Polymer type could influence the rates of these processes as already evident from different vertical distribution pattern for PE, PP and PS debris. The overall persistence of PE, PP and PS in the upper mesopelagic is, however, unclear, as the abundance of these polymers in the abyssal ocean and at the seabed are yet to be measured on relevant spatio-temporal scales.

Our depth-resolved, polymer-specific data set demonstrates that (i) the smaller-sized microplastics were severely underestimated in previous assessments of marine plastic pollution and (ii) considerable amounts of small microplastics are lost from the surface waters and stored in the ocean interior. Focussing on the Atlantic Ocean and including polymer-specific microplastics in size range 32–651 µm, we can now reconcile the existing conundrum of missing ocean plastics^7,10^. We acknowledge that the differences in the particle size and plastic types (bulk vs. specific polymers) of the input and stock terms need to be minimised to allow for their direct comparison. The uncertainties of our data due to the size limit of particles measured and conversion of 2D images into mass concentrations also need to be reduced. We also note that the spatial and vertical trends of plastic abundance and distribution in the Atlantic is likely to vary due to different routes of supply and removal of plastics, which are yet to be constrained. However, despite these limitations, the fundamental conclusion about the Atlantic plastic budget will be unchanged as our estimates are a very conservative minimum estimate of the total mass contamination level.

Based on the plastic waste generation trends from 1950 to 2015 (ref. ^19^) and assuming that the Atlantic Ocean was consistently receiving 0.3–0.8% of the global plastic waste^4^ for 65 years, we estimate the Atlantic waters and sediments to hold 17–47 million tonnes (MT) of plastic litter (‘Methods’ and Supplementary Table 2).

**Fig. 5:**
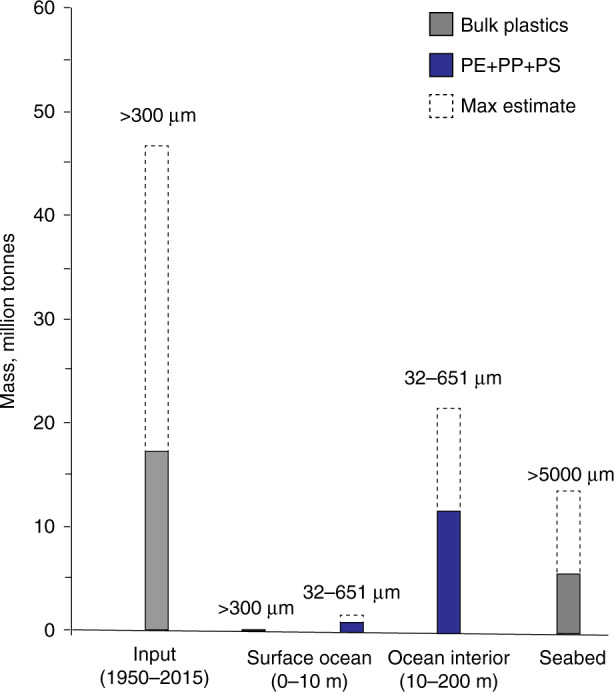
Plastic mass balance for the Atlantic Ocean. Grey bars show the inputs and oceanic inventory of bulk plastic litter from the literature^2,4,7,31,39,60^. Blue bars show the combined mass of polyethylene (PE), polypropylene (PP) and polystyrene (PS) microplastics measured in this study (the most conservative estimates; see Supplementary Methods for details). The upper (max) estimates for each category are shown with dashed bars. Numbers above the bars indicate particle size category. Plastic input is represented by the cumulative mass of bulk plastic litter that is estimated to have entered the Atlantic Ocean between 1950 and 2015 (refs. ^2,4,19^). The load of plastics in the surface ocean (0–10 m) includes the estimated mass of bulk plastic debris (0.1 million tonnes; refs. ^7,39^; Supplementary Table 2) and the combined mass of PE, PE and PS determined in this study (0.8–1.6 million tonnes).

Averaged over all locations and depth layers sampled in this study, the mass concentrations of the investigated polymer-specific microplastic in 32–651 µm size category were 389–719 µg m^−3^ (PE), 216–324 µg m^−3^ (PP) and 58–95 µg m^−3^ (PS). Assuming that these concentrations were representative of the whole area of the Atlantic Ocean^59^ and down to 200 m depth (mean depth of the mesopelagic layer sampled in this study), we calculate the combined weight of these three polymer groups to be 11.6–21.1 MT (PE = 6–14 MT, PP = 4–5 MT and PS = 0.95–1.6 MT) (Fig. 5). This is a significant contribution to ~0.1 MT of larger plastic debris (>300 µm) predicted to be dispersed in the surface waters of the Atlantic^7,39^ and accumulated on the seafloor (5.6–13.5 MT of plastic debris >5 mm in size; ref. ^60^) (‘Methods’ and Supplementary Table 2). Note that the contribution of polymer-specific microplastics of 300–651 µm size category was negligible given their low abundance in the samples (<5%). The high quantities of small polymer-specific plastics that we estimate to be stored in the upper 200 m of the Atlantic are therefore staggering, given that they represent only 5.3% of the Atlantic Ocean volume^59^ and do not include 44% of other littered plastic types^19^ and microplastics in size category below the detection limit of this study^9,33,61^ including nanoplastics^62,63^. Our basin-scale estimates also do not account for the plastic litter that could have been advected to the coastlines^44^ or large quantities of small plastic debris that have already been buried in the deep-sea sediments (e.g. ~80% of plastic particles reported in the Arctic sediments were in ≤25 µm size category^9^).

Including the loads of small microplastics of only three polymer types accumulating in upper mesopelagic into the previously calculated marine plastic stocks can now balance and, given the aforementioned limitations, exceed the cumulative supply of plastic to the Atlantic since 1950s.

Our results demonstrate that there is no missing sink of oceanic plastic; rather, previous assessments of plastic pollution in the ocean were insufficient with respect to particle size collected and water layer surveyed. They also reveal a critical importance of very small, sub-surface microplastics for the oceanic plastic burden, especially relative to larger-sized plastic debris floating in the surface or deposited on seabed. Importantly, our observations are incompatible with the previously calculated supply rates of plastic to the ocean and we conclude that the latter are substantial underestimates. There are several explanations that could be put forward. First, the estimated amount of land-based plastic inputs are fundamentally based on country-scale waste generation data for which a fraction is assumed to enter the ocean^4^ but has not yet been measured directly. Refs. ^2,11^, however, used measurements of microplastic concentrations in rivers to calibrate model coefficients for the plastic inputs via rivers. Further, none of the models account for atmospheric^3^ and maritime inputs of plastics. Most importantly, they completely underrepresent microplastics <300 µm and thus do not account for microplastics that were deliberately produced in microscopic size (e.g. microbeads) and that fragmented/degraded to smaller sizes prior to entering the marine environment. Recent studies demonstrate that microplastics that are directly released into the environment as small plastic particles (<5 mm) could be a significant source of plastic in the ocean^64–66^. The modelled estimates of the global supply of the manufactured microplastics to the ocean are on the order of 0.8–2.5 MT year^−1^ (ref. ^67^), but they are highly uncertain and require validation with empirical data.

The question of missing plastic sinks in the ocean^7,10^ stimulated adoption of better analytical tools, which enabled reliable and unbiased detection of small microplastics in the marine environment. Similar to our methodology, the FTIR imaging that scans the entire filtered particle sample allowed ref. ^33^ to detect 2–3 orders of magnitude more microplastics >11 µm in the Arctic sea-ice compared to the previous assessments based on techniques that require manual pre-selection of potential plastics^68^. As our ability to sample and detect smaller-sized plastics in the ocean improves, the critical need emerges also to constrain robustly the inputs of plastic into the ocean from terrestrial and marine sources, ensuring that small size categories of plastics, including nanoplastics, feature in future field observations and predictive models.

Plastic plays an important role in our lives, providing enormous benefits and savings with respect to health and safety, resource and energy consumption, CO_2_ emissions and production costs^69^. These features cannot currently be matched by any other material^70^. Yet, the durability of the material that is such an advantage in its use is also a cause for concern when plastic is released into the wider environment due to poor waste management practices. To date, a key uncertainty has been the magnitude of contamination of the ocean and our findings demonstrate that this is much higher in terms of mass than has been estimated previously. As plastics are likely to be widely used for many years to come^71^, the need to quantify this material in terms of its sources, sinks and the processes responsible is a matter of considerable urgency. Without this fundamental knowledge, evidence-based conclusion about harms associated with exposure to plastics as well as decisions about the ways society produces, uses and disposes of this very valuable and extraordinary material will not be possible.

## Methods

### Contamination prevention

All steps involving sample collection, processing and analysis were performed in the air-controlled environment: at sea, all the work was done in the laminar flow cabinet (P-range, Bassaire, UK); on land, samples were handled in ISO-5 clean laboratory and in the laminar flow cabinet constructed with laminated high-density non-particle shedding board (Felcon, UK). If not stated otherwise, all laboratory ware was made of glass or stainless steel and cleaned thoroughly prior use (Supplementary Methods). The exposed surface area of the containers was also minimised (e.g. conical flasks were used instead of beakers). All filter membranes used in this study were pre-combusted at 500 °C (stainless-steel and glass-fibre filters) and at 300 °C (silver filters) in a glass petri dishes for 24 h and kept covered at all times. The use of SAPs for particle collection offered significant advantages with respect to preventing air-borne contamination^72^. The sample mesh was locked in the polyvinyl chloride (PVC) filter housing with an outlet (*Ø* 2.5 cm) of the same polymer type, white PP baffle separating the filter mesh of different sizes, butyl O-rings sealing the internal assembly^72^ and stainless-steel clamps locking all the units. Filter housing and all its components were thoroughly cleaned prior to every deployment under the laminar flow hood (Supplementary Methods). Sample mesh was loaded into and removed from the filter housing in the laminar flow cabinet. The filter housing was covered with aluminium foil to exclude any air-borne particles until the last minute before the deployment and during the transit into the ship’s laboratory after the deployment. All chemicals used for sample processing (potassium hydroxide and ethanol) were filtered through a pre-combusted silver filter (nominal pore size 0.8 µm, Sterlitech, USA).

### Sample collection and processing at sea

Water column samples were collected aboard *RRS James Clack Ross* during the passage of the Atlantic Meridional Transect cruise AMT26 JR16001 (http://www.amt-uk.org/) in September–November 2016. At each station/depth, the in situ SAPs (Challenger-Oceanic Ltd.) were used to collect large volumes of seawater (507–1534 L over 50 min of pumping) to capture both suspended and sinking particles^21,22^ onto pre-cleaned stainless-steel mesh with 55 µm aperture size (seawater-resistant, molybdenum bearing grade SS316; The Mesh Company, UK) and nylon 6,6 NITEX mesh with 1 µm aperture size (Sefar, Switzerland). Post-filtration, each particle-loaded mesh was carefully folded and stored at −20 °C until analysis^31^.

### Sample preparation for FTIR imaging

Particles on each mesh were rinsed off using 1 L of artificial sea water (ASW) and each fraction was collected into individual glass flask. The ASW (target concentration 35 g L^−1^) was prepared by first dissolving high-grade pre-combusted salt (500 °C for 24 h) in MilliQ and then filtering the ASW solution through a glass-fibre filter (nominal pore size 0.8 µm, GF/F, Whatman). Individual ASW–particle mixture was split into four fractions of equivalent volume using a Folsom splitter made of polymethyl methacrylate (Plexiglas/Perspex). The split of each size fraction was swirled gently to homogenise the water–particle mixture, and a 100 mL aliquot was immediately transferred into a graduated 200-mL glass flask, covered with a watch glass and incubated with 15 mL of 47% KOH (Merck, Germany) at 60 °C for 72 h to remove particulate organic material^27,28^. Post-digestion, the samples each size fraction was filtered onto a 25-mm stainless-steel filter disc (25 µm aperture size; The Mesh Company, UK) and rinsed with MilliQ to remove salt and with 30% (v/v %) ethanol (Merck, Germany) to reduce surface tension^9^. The particle-loaded filter discs were placed in the glass petri dishes and with their lids opened slightly dried in the laminar flow cabinet for 24–48 h before being fully capped. The remaining filtrate (1–25 µm particle size fraction, which is not a part of this study) was filtered onto silver filters (0.8 µm nominal pore size; Sterlitech, USA), dried and stored until analysis.

### Measurements with FTIR imaging system

The chemical composition of the extracted marine particles was determined using a linear-array FTIR imaging system. The equipment used was Spotlight™ 400 FTIR Imaging System coupled to Frontier™ IR Spectrometer (PerkinElmer, Llantrisant, UK) and equipped with a triple Cassegrain optical system and a 2 × 8 linear array Mercury Cadmium Telluride (MCT) detector. The front and the sample stage of the imaging microscope were surrounded with the Spotlight atmospheric enclosure made of Plexiglas, which minimises atmospheric effects and vibrations and, importantly, the air-borne contamination of the samples and the optical components (Supplementary Fig. 4). The region of interest with a total area of 6 mm × 6 mm, corresponding to 18% of the filtered sample (total area 201 mm^2^ based on the aperture of the Advantec Millipore filtration cup), and positioned over the centre of the filter disc, was scanned in transmission mode over spectral range of 4000–750 cm^−1^ at 8 cm^−1^ spectral resolution and 25 µm pixel resolution applying 4 co-added scans. The boundaries of total area imaged were limited to the aperture of the sample holder (*Ø* = 10 mm) onto which the sample filter was mounted for transmission measurements. For consistency and to prevent sample loss, potential contamination and double-counting of particles, the filter was not repositioned to allow for its other areas to be exposed for further imaging. The imaging area was also binned into 2 × 2 square selections termed markers with area 3 mm × 3 mm each to optimise the spectral data output per image. The IR image background was collected in air under the same spectral settings but with an increased number of co-added spectra (*n* = 120). Each measurement took 88 min and generated 57,600 single spectra over the total area imaged.

The analysis of the acquired hyperspectral IR images and polymer identification was performed using the PerkinElmer Spectrum™ IMAGE and Spectrum™ 10 software. The detailed procedure is described in Supplementary Methods. Briefly, chemometric technique of principal component analysis (PCA) was used to first explore the chemical composition of the entire imaged particle sample and collect spectra from each variation (score) displayed on the reconstructed PCA-based IR image^73–75^ (Supplementary Fig. 5a, b). The collected individual spectra were then exported into the Spectrum™ 10 software (Supplementary Fig. 5c) for identification via comparison against the spectra in the reference polymer library (18,711 polymer types; spectra database from S.T. Japan-Europe GmbH, Germany/Japan). Spectra with the hit quality >0.7 (max. hit score of 1) were accepted as verified polymers types^9,76^ and saved. From those, the best quality spectra for PE, PP and PS were selected and used as reference spectra to plot correlation maps against every pixel constituting the IR image using Spectrum™ IMAGE (Supplementary Fig. 6a). In essence, the reconstructed IR image was showing the locations of and areas occupied by each of the examined polymer type. These polymer-specific IR images were then exported into the FIJI Image J image software for particle count and characterisation^77,78^ (see Supplementary Methods, Supplementary Fig. 6b and Supplementary Table 4).

### Quantifying number concentrations of microplastics

Polymer-specific particles were counted in each of the four 3 mm × 3 mm markers making up the total imaged area (6 mm × 6 mm) of the filter. For each sample, we treated the four markers as sample replicates^24^ to account for the uneven distribution of plastic particles on the sample filter, similar to the method in ref. ^33^. We calculated the mean and standard deviation of the four replicates to determine particle count of the imaged area. These values were then scaled to particle per unit volume units (m^−3^) using known total filter area (201 mm^2^), volume of a sample fraction/split filtered for FTIR imaging and the total volume of seawater collected with in situ pump per each sample (Supplementary Data 1; see similar approach in ref. ^3^). Error propagation for calculation of particle number per filter included the error from the conversion of full sample volume to the investigated sample fraction (split). The former was based on the practical accuracy of the SAP flow rate, which is ~±2% for a flow rate of 60 L h^−1^ for clogged filters through to 1000 L h^−1^ in clear waters (Challenger Oceanic Ltd.). The error from splitting the water–particle mixture was estimated to be ~5% based on the gravimetric measurements of a MilliQ water sample split in the same manner as the samples. The error associated with the measurement of the size of the filtered and scanned areas were considered negligible.

### Procedural blanks

The unused meshes were prepared and processed on board of the ship in exactly the same manner as the samples. For the blank test, 3 L of MilliQ water were passed through the clean unused meshes (in triplicates) loaded into the SAP filter housing in the laminar flow cabinet. Handling of the procedural blanks in the land laboratory in preparation for FTIR imaging was performed in the same way as the samples. The contamination of blanks with PE, PP and PS were examined by reconstructing the acquired IR images of the blank samples against the respective reference spectra identified in the actual samples (Supplementary Table 3). All the polymer spectra selected as reference spectra had a hit score >0.90 (i.e. 90% similarity with the library spectra). No microplastic particles reliably identified as PE, PP and PS were detected in the procedural blanks (Supplementary Figs. 1 and 2). Suspect pixels with elevated correlation coefficients (0.615–0.634) against reference polymer spectra were found in blank #2/marker 2 and identified as polymethyl pentane (PMP; spectrum ID SP0061; best hit score = 0.78) and in blank #3/marker 4 and identified as benzyl butyl phthalate (BBP; spectrum ID AD0232; best hit scores = 0.91). PMP is a rigid polyolefin and was identified in 5 samples out of 36 with a hit score range 0.73–0.83. BBP is a common plasticiser for PVC and was present in 4 samples with a hit score range 0.86–0.97 (Supplementary Data 2). BBP spectra were present in other 14 samples, although their quality was below acceptable (hit score range 0.47–0.69; Supplementary Data 2). BBP plasticiser is a sticky liquid that could have been released from the PVC-based SAP filter housing or from the PVC particles themselves during digestion procedure and stuck on the mesh wire. The locations of these polymer types on the IR image maps did not coincide with any of the polymer groups of interest.

### Mass conversion

The mass of the individual microplastic particles was calculated by multiplying their volume by their polymer-specific density (Supplementary Data 1). Particle volume was estimated using the particle dimensions (length and/or area) measured on the IR image and assuming a range of particle shapes as applied in previous studies (refs. ^10,29,31,32^). Here we report the most conservative estimates (Method IV/Flake), which were obtained using the method of ref. ^10^. The details and sensitivity analysis for mass conversions using all the applied methods are provided in Supplementary Methods and summarised in Supplementary Fig. 3. Mass concentrations of polymer-specific microplastics (in µg m^−3^) were calculated by multiplying their average particle mass by number concentration in each sample (Supplementary Data 1).

### Mass budget of plastics in the Atlantic Ocean

The cumulative input of plastic debris to the ocean from 1950 to 2015 and their current loads in different compartments (surface water, water column and deep-sea sediments) of the Atlantic Ocean were estimated using the data available from literature^2,4,7,10,19,39,60^ and including the polymer-specific mass concentrations measured in this study (Supplementary Table 2).

Plastic inputs to the Atlantic Ocean were calculated using the estimates of the global plastic waste generation from 1950 to 2015 (ref. ^19^; Supplementary Table 2). The fraction of the global plastic waste, by weight, that has entered the Atlantic by 2015 was calculated by first considering the estimates of mismanaged plastic waste generated in and available to be discarded only from the countries bordering the Atlantic Ocean in 2010 (refs. ^2,4^; Supplementary Table 2). The sources included mismanaged plastic waste from the coastal areas (>5 mm in size; ref. ^4^) and rivers (>0.3 mm in size; ref. ^2^). Note that ref. ^2^ obtained river inputs using Jambeck et al.’s mismanaged plastic waste model^4^, river catchment areas and locations of artificial dams, and hence these inputs are not additional but have been included in Jambeck et al.’s estimates for 2010 (ref. ^4^). Further, the assumption was made that for the given period, the fraction of the global plastic waste entering the Atlantic Ocean every year was similar to that in 2010 (0.3–0.8%; ref. ^4^).

Plastic stocks in the Atlantic Ocean were based on the mass load of buoyant plastics in the Atlantic sea-surface reported in refs. ^7,39^ (also references therein) and based on the data from net/trawl surveys in combination with large-scale surface ocean circulation models (Supplementary Table 2).

Due to the scarcity of data on plastic accumulation in the deep-sea sediments, their loads in global and regional scales are currently very crude. Ref. ^60^ quoted the load plastic debris of >5 mm size category on the global seabed being on the order of around 25–65 MT; we scaled these values to the area of the Atlantic Ocean (81.2 × 10^6^ km^2^, excluding the Baltic and the Mediterranean Seas; ref. ^59^). The resulting Atlantic-wide sedimentary loads must be considered in light of the uncertainties associated with variable distribution of plastic litter on the seafloor due to different processes responsible for their supply, removal, transit, degradation and redistribution in the marine environment.

Basin-wide extrapolation of the abundances of polymer-specific microplastics measured in our study is described in the main text. The range of stocks reported for each polymer type results from the assumption of different particle shapes when converting concentration units from number to mass (see relevant ‘Methods’ section and Supplementary Information).

### Statistical analyses

The statistical comparisons are based on nonparametric statistics (Mann–Whitney *U* test) and performed using the open source R software (version 3.4.1; https://www.r-project.org/). The significance level was chosen to be 1% (*α* = 0.01).

### Reporting summary

Further information on research design is available in the Nature Research Reporting Summary linked to this article.

## Supplementary information

Supplementary Information

Peer Review File

Description of Additional Supplementary Files

Supplementary Data 1

Supplementary Data 2

Reporting Summary

## Data Availability

All polymer-specific data underlying this study can be downloaded from the British Oceanographic Data Centre (BODC; 10.5285/aadd4168-0398-14f5-e053-17d1a68b059d and ref. ^24^) or are available from the corresponding author upon request. The Argo data of monthly mixed layer climatology under product name ‘May 2018 netcdf format’ were downloaded from the Argo Mixed Layer website (http://mixedlayer.ucsd.edu/) hosted by Scripps Institution of Oceanography, UC San Diego. The Argo data used to create this climatology were collected and made freely available by the International Argo Program and the national programmes that contribute to it (http://www.argo.ucsd.edu, http://argo.jcommops.org). The Argo Program is part of the Global Ocean Observing System (10.17882/42182#56126). The CTD profile data collected during the AMT26 JR16001 expedition were provided by and are freely available from BODC (10.5285/aa51baf6-2095-6c28-e053-6c86abc0d7f7 and ref. ^23^).
