# Supplementary material for: High concentrations of plastic hidden beneath the surface of the Atlantic Ocean
Source: Nat Commun. 2020 Aug 18;11:4073. doi: 10.1038/s41467-020-17932-9 (PMC7434887; doi:10.1038/s41467-020-17932-9)
Supplement: Supplementary file 1 — Supplementary Information [file 41467_2020_17932_MOESM1_ESM.pdf]

Supplementary Information for "High concentrations of plastic hidden beneath the surface of the Atlantic Ocean"

Katsiaryna Pabortsava[1,*] and Richard Lampitt[1]

[1] National Oceanography Centre, European Way, Southampton, SO14 3ZH, UK

Supplementary Methods

Cleaning laboratory ware and filters

The SAP filter housing and all its components (outlet and baffle) were cleaned with 10% hydrochloric acid (HCl; Fisher Chemical, UK) and thoroughly rinsed with MilliQ water (resistivity 18.2 MΩ×cm; equipped with 0.22 µm Millipack® membrane filter) prior to each deployment. Conical glass flasks and the watch glasses (all Fisherbrand, UK) used for digestion were cleaned with 50% (v/v%) HCl at 130°C followed by a thorough rinse with MilliQ water (the final rinse was always done in the laminar flow cabinet) and dried on a sheet of cotton Twill wipe (Contec©, Canada) in the laminar flow cabinet. Other glassware (e.g. petri-dishes, filtration funnel, measuring cylinders, syringe) were soaked in 10% HCl solution, thoroughly rinsed with MilliQ and dried in a laminar flow cabinet. The components of the Folsom splitter were cleaned in a similar manner.

The 30 cm stainless-steel filter disks (SS316 Grade TWILL Woven Wire Mesh #300 with 55 µm aperture; The Mesh Company; UK) for SAPs were rinsed with MilliQ, wrapped in a double layer of aluminium foil and combusted at 500°C. The 30 cm nylon 6,6 NITEX mesh circles (1 µm aperture size; Sefar, Switzerland) were soaked in 10% (v/v%) HCl overnight, rinsed off thoroughly with MilliQ, and wrapped into a double layer of pre-combusted aluminium foil. The stainless-steel filter disks for post-digestion filtration (Ø=25 mm; SS316 Grade TWILL Woven Wire Mesh #500 with 25 µm aperture; The Mesh Company; UK) and glass-fibre filter disks (Ø=25 mm; nominal pore size 0.8 µm; Whatman) were combusted in a similar manner but in a clean glass petri dish (Duran, Germany) which was also wrapped in a double layer aluminium foil. Silver filter disks (Ø=25 mm, nominal pore size 0.8 µm; Sterlitech) were soaked in 50% (v/v%) HCl for 24 hrs, then thoroughly rinsed with MilliQ and combusted at 300°C in a glass petri dishes (Duran, Germany) wrapped in double layer of aluminium foil.

FTIR image processing

The IR image generated by FTIR scanning represents a 3-D hyperspectral data cube, which combines spatial information of imaging system and spectral information of the FTIR spectrometry. In essence, each pixel of an IR image contains a spectrum over a certain wavelength range (here, 4000-750 cm^{-1}), and thus allows for visualisation of the chemical composition of the sample surface [1, 2, 3]. The data-rich IR images require conversion into a set of signals (2-D data matrix) which can then be analysed using classical chemometric and noise reduction techniques, such as principle component analysis (PCA)[1, 2, 3].

We followed 4 steps to process and analyse the acquired IR images:

Step 1: Noise removal and data exploration with PCA using PerkinElmer Spectrum™ IMAGE software.

Step 2: Spectral identification and classification using PerkinElmer Spectrum™ 10 software.

Step 3: IR image reconstruction using polymer-specific spectra using PerkinElmer Spectrum™ IMAGE software.

Step 4: Particle quantification and characterisation using FIJI-Image J software[4].

Noise removal and data exploration (Step 1) were performed using the toolboxes in Spectrum™ IMAGE software. Following acquisition, each spectrum in the IR image was corrected for the interference of water and CO_2 bands using 'Atmospheric correction' function (Supplementary Fig. 5a). The background spectrum was removed automatically during the scan. The spectral variations in the IR image were investigated using PCA[3, 5, 6] embedded into 'Show Structure' function of the Spectrum™ IMAGE (Supplementary Fig. 5b). In essence, PCA is a chemometric and noise-reduction tool that compares every spectrum of the IR image to the average spectrum and shows how much of each kind of variation (termed score) is present

in each spectrum. The PCA algorithm extracts 20 most significant principal components (scores). Automatic noise weighing was used to account for variations in the source power across the spectral range. The PCA-based IR image was then reconstructed from the significant scores displayed, while noisy scores were excluded. Individual spectra in each score were then collected and saved.

Spectral identification and classification (Step 2) was performed by loading the saved spectra in batches of 100 into the Spectrum™ 10 software (the size of the batch depends on the computational abilities of a computer) (Supplementary Fig. 5c). Each spectrum was then compared against the spectra in the reference library (18,711 polymer types; spectra database from S.T. Japan-Europe GmbH, Germany/Japan). The identification was accepted as successful when the similarity (here as best hit score from 0 to 1) between the measured and the library spectrum was ≥ 0.7 (equivalent to 70% in refs[7, 8]). The polymer type was assigned to a spectrum on the basis of the highest hit score. The *x* and *y* coordinates of all successful spectra on the IR image, their hit scores, library search reference and polymer type description were saved into a spreadsheet. For each sample marker, the best hit spectra corresponding to the types of polyethylene, polypropylene and polystyrene were then selected and grouped (Supplementary Table 3).

Polymer-specific IR image reconstruction (Step 3) was performed using 'Compare correlation' function in Spectrum™ IMAGE (Supplementary Fig. 6a). For each polymer type a spectrum with the highest hit score was assigned as a reference spectrum and imported back into Spectrum™ IMAGE to indicate the areas on the image where the spectra were the most similar to that reference spectrum (Supplementary Fig. 6a). Knowing the part of an image that contained a particular substance and having a reference spectrum to that substance, enabled the determination of the exact area where a particulate substance was most prevalent. We used a correlation coefficient of 0.7 to display the areas of polymer-specific particles on the

reconstructed IR image (Supplementary Fig. 6a). This allowed to account for the range of the successful hit scores and for some deterioration of the signal towards the edge of a particle[9].

Polymer-specific microplastic quantification and characterisation (Step 4) were performed in FIJI package of ImageJ software[76]. All polymer-specific IR images were first converted to 8-bit images and then overlaid with respect to their polymer group and marker ID (Supplementary Fig. 6b). Note that in this way we generalised polymer types into a broader polymer group (Supplementary Table 3). The combined images were then converted into binary images with a threshold for pixel brightness set manually for every image to optimise the accurate representation of the particle area (see FIJI ImageJ macro script in Supplementary Table 4 and also ref[10]). Binary images (Supplementary Fig. 6b) were then run through a macro (Supplementary Table 4) which included 'Analyse Particles' function, providing counts of particles per image and for individual particles, the measurements of area, horizontal dimensions (e.g. lengths of minor major axes, minimum and maximum Feret diameters) and morphological characteristics (e.g. circularity and roundness).

Estimating volume and mass of microplastic particles

From the polymer-specific IR images, the information on the apparent particle surface area and two horizontal dimensions of particles were generated. The third dimension of particles (thickness or height) was thus required to first calculate particle volume and then their mass by multiplying volume by polymer-specific density (PE=0.880-0.960 g cm^{-3}; PP=0.895-0.92 g cm^{-3}; PS=0.960–1.04 g cm^{-3})[11]. The area of particles and their major and minor dimensions were measured on the IR image using Fiji Image J software[4]. Particle area A was determined as the sum of all square pixels (in calibrated units of μm^2) making up a particle. Horizontal dimensions (length and width) were described by fitting an ellipse with major (L) and minor (l) axes.

In our study we estimate particle volumes following four approaches:

Method I – Sphere. We transformed the irregularly shaped particles into circles with areas equivalent to their apparent square pixel-based areas *A*. Therefore, the volume of each particle was estimated as $V_1 = \frac{4}{3}\pi R^3$, where radius was $R_1 = \sqrt{A/\pi}$ (e.g. refs[12, 13, 14]). Note that with this method we did not make an assumption of particle shape. Specifically, we did not assume that the particles were spheres – rather, we transformed/reshaped the apparent surface area of particles into circles. This method also did not rely on the horizontal axes or shape particularities as we solely utilised the apparent surface area. Finally, since particles were reshaped into circles, an additional assumption about the particle orientation was not required when extrapolating to the third dimension (i.e. thickness/height) – spheres are invariant to rotation.

Method II - ellipsoid: we followed the method for particle volume estimation described in refs[9, 11]. This method explicitly assumes that the particles were of ellipsoid shape with semi-axes *L/2, l/2, h/2,* area $A_2 = \pi Lh$ and volume $V_2 = \frac{1}{6}\pi lLh$, where *h* is the estimated thickness/height. Method II is sensitive to horizontal axes of 2D representation of the particles. When transforming the shape of the particles into ellipses, the area is not necessarily preserved as new areas are included and other are excluded, especially if the original particle shape has a lot of irregularities. When tested with our polymer-specific particle dataset, this method only slightly underestimated the surface area of the particles relative to the apparent surface area (mean difference was 99.95%), indicating that the observed shapes of the particles were ellipsoids in 2D. Method II required extra assumption when extrapolating to third dimension (*h*) based on the particle aspect ratio. For example, Poulain et al. 2019 assumed that *h* was of the equal size to the minor axis[11]. In contrast, Simon et al. 2018 assumed that the aspect ratio between *h* and *l* was equivalent to the ratio of *l* to *L* (ref[9]). Simon et al. used the median aspect

ratio for the entire particle dataset[9], while Poulain et al. considered individual aspect ratios of particles when extrapolating to the third dimension[11]. The method in Simon et al. is sensitive to historical data and is unsuitable for comparison with our datasets[9]. The method in Poulain et al. was thus preferred for comparison[11].

Method III - cylinder: the particle volume was described following ref[15]. This method explicitly assumes that the particles were flat cylinders with base area A_3 and height h_3. Similar to Method I, this approach utilised the apparent square pixel-based area A. The assumption of height h_3 was made preserving the minor axis (i.e. $h_3=l$). Therefore, the particle volume was calculated as $V_3=A\times l$.

Method IV - flake: the particles volume was described following ref[16]. This method assumes that all particles are flat fragments or flakes with a nominal length L, corresponding to the length of the major axis, and a factor $\alpha=0.1$, corresponding to a flat shaped volume. The volume V_4 of the particle was derived as $V_4=L\times\alpha$.

The distribution and average particle volume in the tested particle set (n=1444) is shown in Supplementary Fig. 3. The average particle volumes were of the same order of magnitude with the average difference between the highest (Method II – cylinder) and the lowest (Method IV-flake) volume estimates being a factor of 2.

The influence of density on mass was negligible compared to the choice of volume. In our calculations the following specific polymer densities were used: PE=0.975 g/cm^3, PP=0.946 g/cm^3, PS=1.039 g/cm^3.

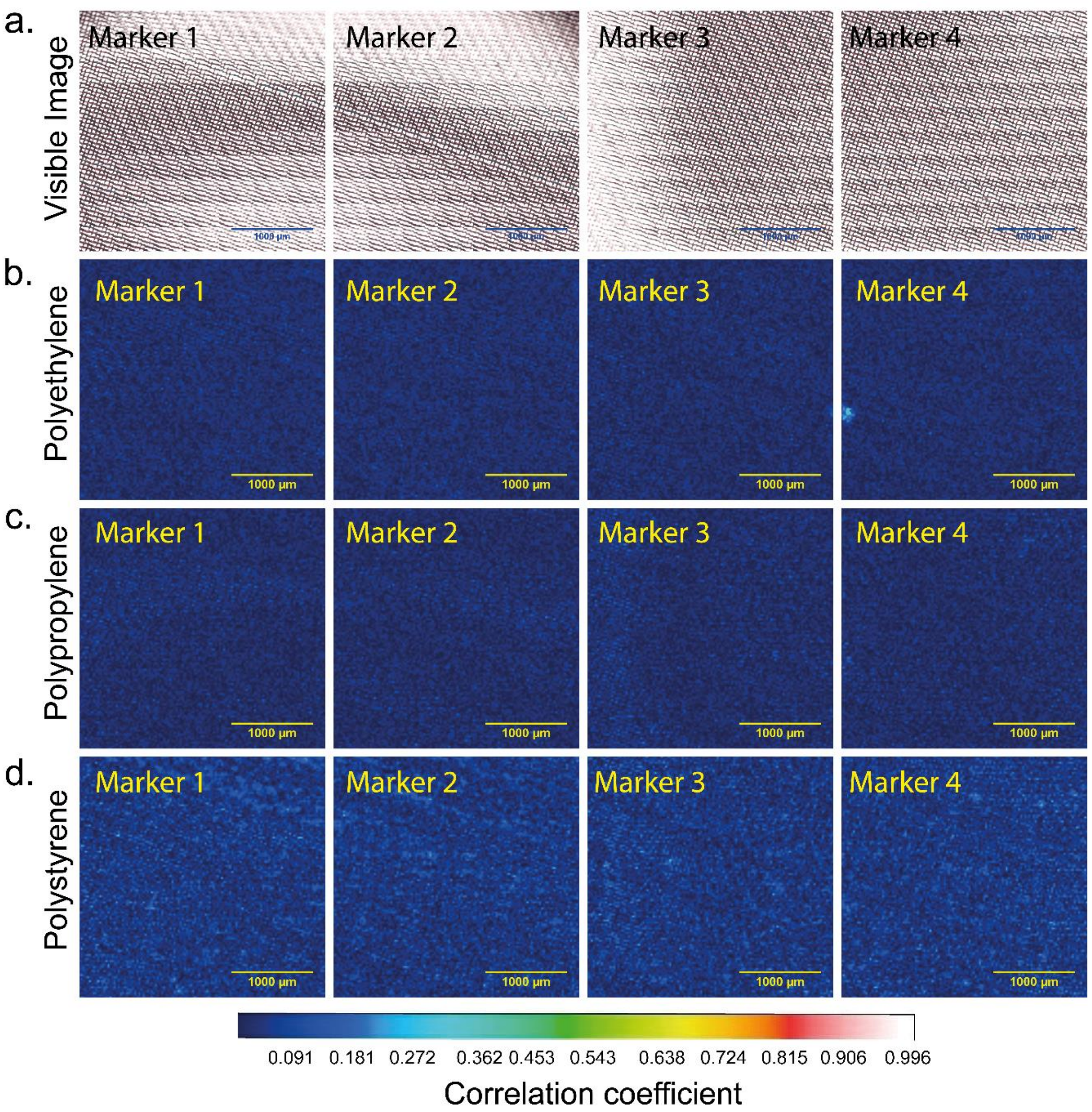

Supplementary Figure 1| Example of the test for microplastic contamination in the procedural Blank #1. a, visible image of each marker on the blank filter mesh (3 mm ×3 mm). **b-d,** the IR image of each marker plotted as a correlation map against the reference polymer spectra of **b,** polyethylene (HU0708), **c,** polypropylene (HU0566) and **d,** polystyrene (HU0647) (also in Supplementary Table 3) commonly found in the actual particle samples. Each marker was imaged once with 4 co-added scans per pixel (other settings are detailed in Methods). All images were generated in PerkinElmer Spectrum™ IMAGE software; scale is 1000 µm.

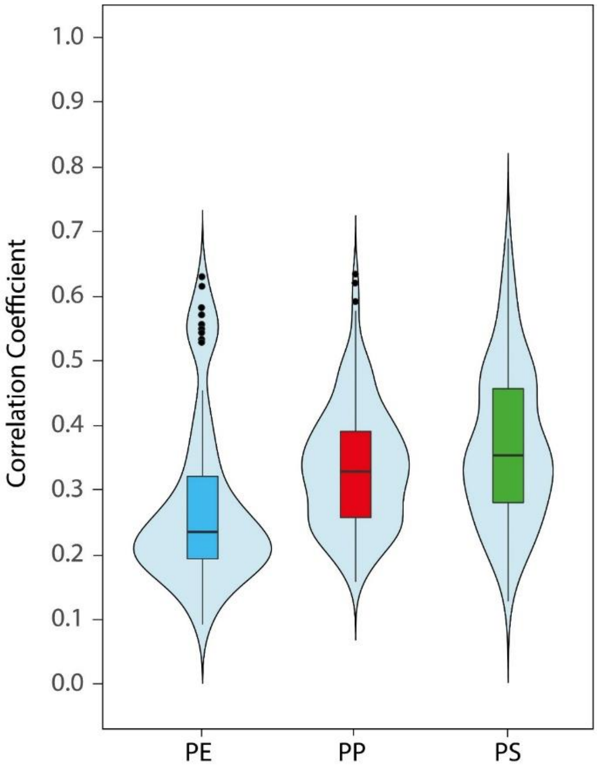

Supplementary Figure 2| Summary of the test for polymer-specific microplastics contamination in the procedural blanks. The IR images of triplicate blank samples were reconstructed by plotting every pixel of the IR image against the reference spectra of polyethylene (PE, n=9), polypropylene (PP, n=15) and polystyrene (PS, n=10) found in the actual samples (Methods; Supplementary Table 3). The distribution of maximum correlation coefficients found in each image marker (4 per image) is depicted as violin plots (light blue). The wider sections of violin show higher probability of observations of a given value and the thinner sections correspond to a lower probability. In embedded boxplots, the lower and upper boundaries of each box indicate the 25th and 75th percentile, respectively. Upper whiskers show the largest values within 1.5 times interquartile range above 75th percentile; lower whiskers show the smallest value within 1.5 times interquartile range below 25th percentile. In each box, the median value is shown as a black horizontal line. Black circles indicate outliers.

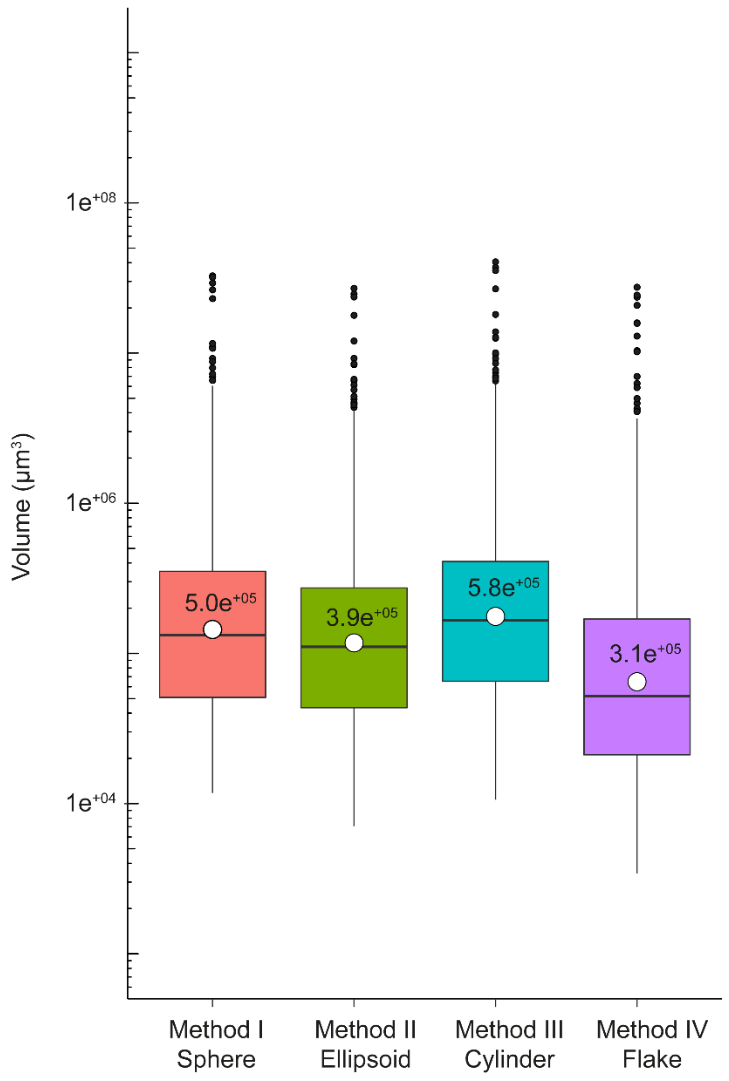

Supplementary Figure 3| Sensitivity test for estimating the volume of microplastic particles. The entire microplastic particle set (n=1444) was used to estimate the particle volume based on their two-dimensional metrics and approaches described in refs[9, 11, 12, 13, 15, 16] (see Supplementary Methods). The lower and upper boundaries of each box indicate the 25th and 75th percentile, respectively. Upper whiskers show the largest values within 1.5 times interquartile range above 75th percentile; lower whiskers show the smallest value within 1.5 times interquartile range below 25th percentile. In each box, the median value is shown as a black horizontal line; white circles with numbers above indicate mean particle volume computed for each method. Black circles indicate outliers.

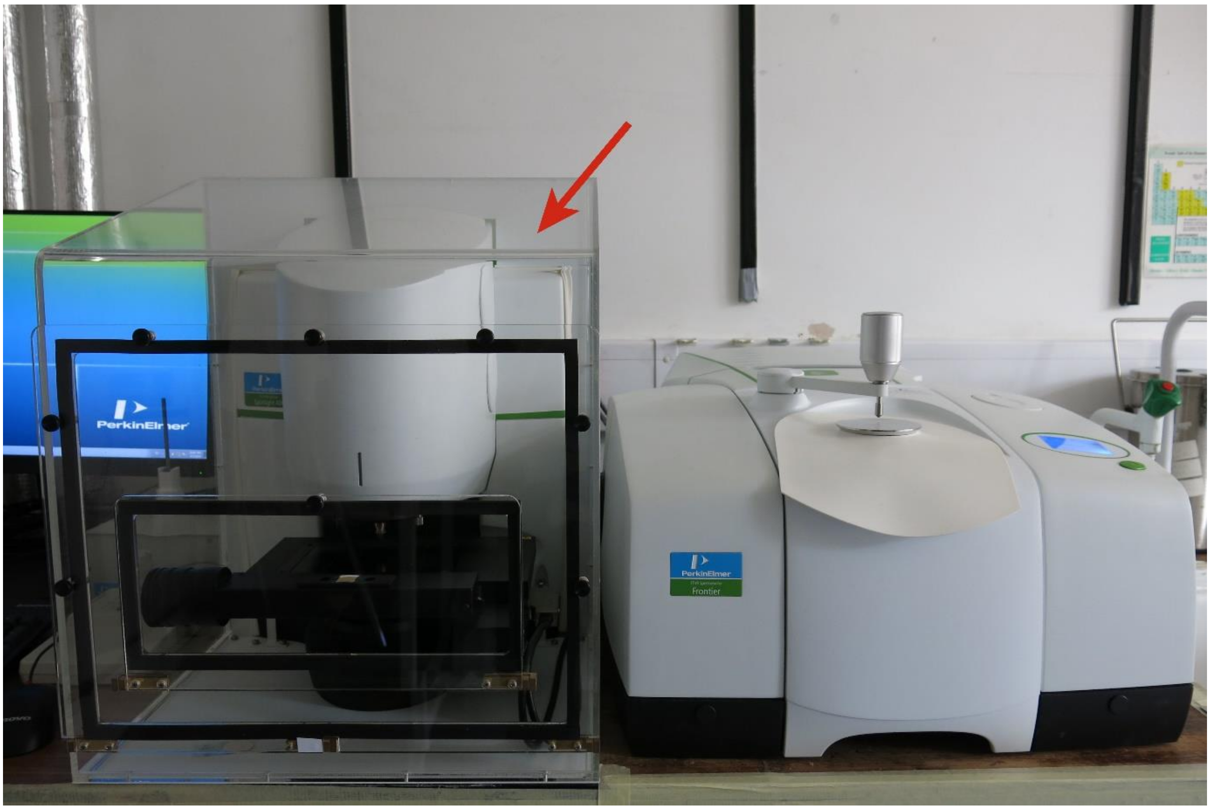

Supplementary Figure 4| PerkinElmer Spotlight 400 FTIR imaging system. A Plexiglas atmospheric enclosure (indicated with a red arrow) was purposefully installed around the Spotlight™ 400 FTIR imaging microscope to prevent air-borne contamination during measurements.

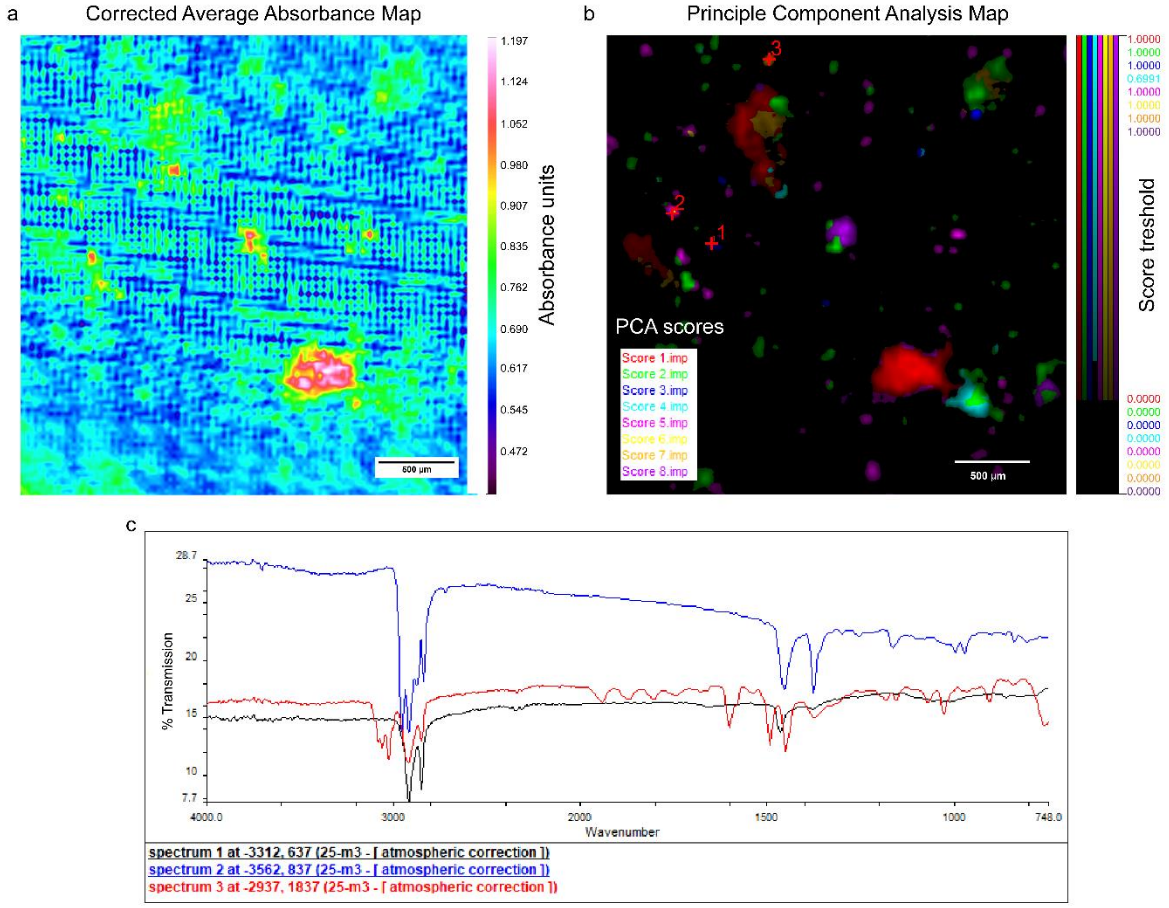

Supplementary Figure 5| Noise removal and data exploration of the IR image. a, The original IR image/map (colour correspond to absorption units) of the 3 mm × 3 mm sample area corrected for atmospheric interference. **b,** the same IR image reconstructed using principle component analysis (PCA) with scores (n=8) displayed. Different colours indicate different PCA scores and their locations on the IR map. Note, that in **b** the area covered by each PCA score displayed is defined by score threshold settings (shown as scale bar from 0 to 1 for each score) and does not represent the true area of the particles. **c,** IR spectra collected from different PCA scores in **b** (numbered red crosses) and exported for polymer identification in PerkinElmer Spectrum™10 software.

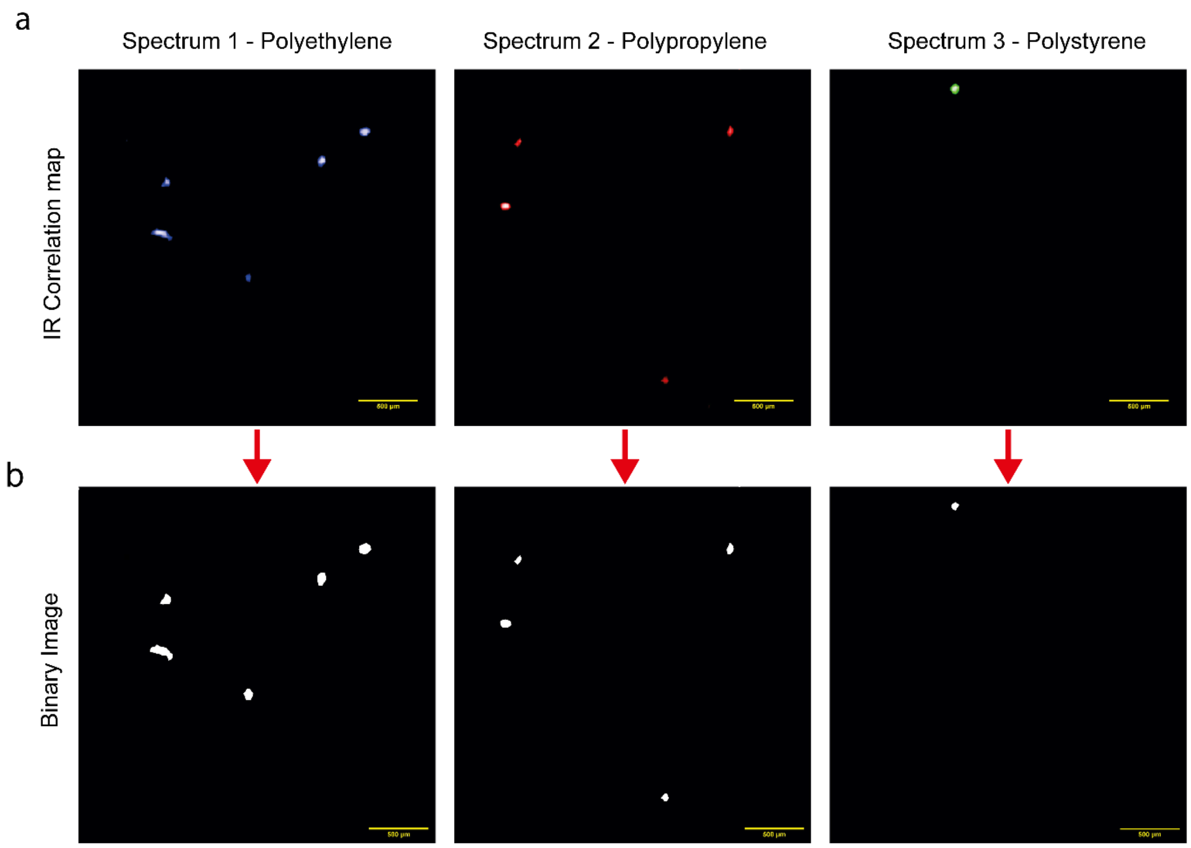

Supplementary Figure 6| Reconstructed polymer-specific images. a, Polymer groups of polyethylene, polypropylene and polystyrene displayed on a false colour IR image using the correlation with the respective reference spectra collected from the PCA map (Supplementary Fig. 5b, c). **b,** polymer-specific correlation maps in **a** converted to binary images (indicated with red arrows) to be analysed and characterised for particle count, horizontal dimensions, and area (all using FIJI Image J software[4]). The IR image used to produce correlation maps represents a single measurement with 4 co-added scans per each pixel (other settings of FTIR imaging are detailed in Methods). The length of the yellow scale bar in **a** and **b** is 500 µm.

Station ID	Sampling date dd/mm/yy	Sampling location		Depth (m)	Layer	Particle counts (N/imaged area)			Number concentration (particles m^{-3})			Mass concentration (µg m^{-3})		
		°N	°W			PE	PP	PS	PE	PP	PS	PE	PP	PS
1	25/09/16	45.8	13.6	10	10 m	NA	NA	NA	NA	NA	NA	NA	NA	NA
3	29/09/16	37.7	24.9	10		21(5±3)	64(16±3)	16(4±4)	1455±759	4435±857	1109±1024	578±582	1152±676	52±70
6	04/10/16	21.2	29.7	10		26(7±2)	12(3±1)	2(1±0)	1247±314	576±184	96±56	367±179	342±125	4±5
8	06/10/16	14.5	28.6	10		87(22±4)	0(0±0)	1(0±0)	6838±1507	0±0	79±79	1253±676	0±0	11±13
9	07/10/16	11.2	27.7	10		44(11±3)	19(5±1)	0(0±0)	2095±583	905±169	0±0	418±115	624±171	0±0
12	10/10/16	1.2	25.3	10		17(4±1)	11(3±1)	12(3±3)	856±153	554±161	604±543	400±296	129±112	1422±1302
15	13/10/16	-9.1	25	10		3(1±0)	17(4±0)	2(1±0)	135±87	765±115	90±53	19±21	301±104	3±3
18	17/10/16	-21.3	25	10		16(4±2)	15(4±1)	9(2±1)	713±448	668±254	401±259	208±251	195±125	125±93
19	18/10/16	-24.5	25	10		20(5±2)	3(1±0)	0(0±0)	900±324	135±87	0±0	437±310	54±89	0±0
23	22/10/16	-37.3	28.4	10		25(6±1)	21(5±1)	3(1±0)	1064±236	931±145	133±86	1327±901	35±38	15±19
27	27/10/16	-50.3	34.1	10		19(5±1)	0(0±0)	0(0±0)	2088±640	0±0	0±0	259±213	0±0	0±0
30	31/10/16	-53.4	41.5	10		24(6±2)	1(0±0)	0(0±0)	1662±611	69±70	0±0	1233±495	1±1	0±0
1	25/09/16	45.8	13.6	60	Intermediate	22(6±1)	2(1±1)	2(1±0)	1621±250	147±148	147±86	617±409	68±84	105±102
3	29/09/16	37.7	24.9	65		28(7±2)	0(0±0)	1(0±0)	2105±582	0±0	100±87	808±495	0±0	39±59
6	04/10/16	21.2	29.7	100		47(12±2)	5(1±0)	7(2±1)	1988±413	212±84	296±130	315±236	54±28	25±16
8	06/10/16	14.5	28.6	50		178(45±14)	42(11±2)	56(14±8)	7541±2425	1779±416	2373±1383	1083±596	201±115	178±141
9	07/10/16	11.2	27.7	50		65(16±4)	0(0±0)	0(0±0)	2860±719	0±0	0±0	999±515	0±0	0±0
12	10/10/16	1.2	25.3	70		49(12±5)	4(1±1)	0(0±0)	2040±800	167±119	0±0	547±274	30±31	0±0
15	13/10/16	-9.1	25	100		17(4±2)	1(0±0)	0(0±0)	649±261	38±38	0±0	216±193	6±10	0±0
18	17/10/16	-21.3	25	170		13(3±1)	0(0±0)	2(1±1)	499±178	0±0	77±77	66±57	0±0	2±4
19	18/10/16	-24.5	25	140		22(6±1)	5(1±1)	0(0±0)	838±209	191±98	0±0	134±114	51±28	0±0
23	22/10/16	-37.3	28.4	100		44(11±2)	6(2±0)	0(0±0)	1685±366	230±50	0±0	224±136	29±20	0±0
27	27/10/16	-50.3	34.1	100		37(9±2)	7(2±1)	1(0±0)	2553±708	483±180	69±69	226±177	89±50	14±16
30	31/10/16	-53.4	41.5	100		0(0±0)	0(0±0)		0±0	0±0	0±0	0±0	0±0	0±0
1	25/09/16	45.8	13.6	100	Mesopelagic	23(6±1)	22(6±2)	3(1±0)	1825±455	1745±639	238±83	118±101	1883±858	14±11
3	29/09/16	37.7	24.9	150		34(9±1)	2(1±1)	3(1±0)	1653±200	97±113	146±58	111±87	38±45	6±12
6	04/10/16	21.2	29.7	200		20(5±2)	1(0±0)	1(0±0)	1075±468	54±54	54±54	312±235	3±5	3±6
8	06/10/16	14.5	28.6	150		29(7±2)	9(2±1)	0(0±0)	1376±342	427±253	0±0	136±107	58±61	0±0
9	07/10/16	11.2	27.7	150		11(3±1)	0(0±0)	3(1±1)	797±331	0±0	217±218	51±42	0±0	10±17
12	10/10/16	1.2	25.3	170		13(3±0)	1(0±0)	1(0±0)	1115±198	86±86	86±86	103±82	3±3	2±4
15	13/10/16	-9.1	25	200		16(4±1)	2(1±0)	0(0±0)	903±183	113±66	0±0	110±45	4±6	0±0
18	17/10/16	-21.3	25	270		6(2±1)	10(3±2)	0(0±0)	448±198	747±455	0±0	60±69	1116±709	0±0

19	18/10/16	-24.5	25	240		8(2±2)	1(0±0)	0(0±0)	520±441	65±65	0±0	73±102	8±16	0±0
23	22/10/16	-37.3	28.4	200		10(3±1)	5(1±1)	0(0±0)	1101±536	551±420	0±0	234±187	32±35	0±0
27	27/10/16	-50.3	34.1	200		16(4±2)	3(1±0)	0(0±0)	1355±744	254±164	0±0	345±213	2550±1727	0±0
30	31/10/16	-53.4	41.5	200		7(2±0)	11(3±2)	0(0±0)	462±81	726±480	0±0	211±227	106±81	0±0

Supplementary Table 1| Abundance of polymer-specific microplastics in the study region. The column on particle counts provides the total number of polyethylene (PE), polypropylene (PP) and polystyrene (PS) particles detected in a total imaged area (6 mm x 6 mm) of each sample. Numbers in parenthesis refer to the number of particles averaged over four (n=4) individual markers (area 3 mm x 3 mm for each) constituting the total imaged area of a sample (data presented as mean± standard error measurement; see ref.[17]). The calculation of particle number and mass concentrations is detailed in Methods and Supplementary Data 1. Mass concentrations values presented here are based on estimates using Method IV (ref[16]; see Supplementary Methods). For number and mass concentrations, error is based on propagated uncertainties and presented here reported as ± standard deviation. No near surface (10 m) sample was taken at 45°N due to pump failure.

	Year/ Period	Size range	Plastic type	Weight (Million Tonnes)		References
				Global	Atlantic Ocean	
Plastic inputs into the ocean						
Plastic waste generated	1950-2015	NA	Bulk	5800	NA	Fig. S4; ref.[18]
Plastic waste discarded	1950-2015	NA	Bulk	4900	NA	Fig. S4; ref[18].
Discharge from land	2010	>300 µm	Bulk	4.8 *(2)* -12.4 *(5)*[*]	0.83 *(0.3)* - 2.21 *(0.8)*[*]	Ref[19, 20]
Cumulative discharge from land	1950-2015	>300 µm	Bulk	117 - 292	17-47	Ref[19, 20]
Oceanic stocks						
Surface (0-0.5 m depth)	NA<br>NA	0.3-200 mm<br>> 200 mm	Bulk<br>Bulk	0.236<br>0.203	0.032<br>0.066	Ref.[21]<br>Ref. [22]
Seabed	NA	> 5 mm	Bulk	25-60	5.6-13.5[**]	Ref.[23]
Atlantic Ocean (10-200 m depth)	2016	>32-651µm	PE<br>PP<br>PS	NA<br>NA<br>NA	6-14<br>4-5<br>0.95-1.6	This study

[*] numbers in parenthesis indicate the percent of the cumulative global plastic waste generated between 1950 and 2010 (274 Million Tonnes, ref[18])

**calculated as a proportion of the area of the Atlantic Ocean to the area of the global ocean (22.5%; ref[24]) (see Methods section for details)

Supplementary Table 2| Summary of the data used to calculate plastic budgets in the Atlantic Ocean.

Polymer type	Library ID	Group
ASTRO TURF/POLYETHYLENE GREEN/STD 416	DA3017	Polyethylene
CTA 87 A0433 POLYETHYLENE	FI0072	
CTA 87 C0453 DOUBLE COATED/BLACK/SILVER POLYETHYLENE	FI0083	
POLYETHYLENE 8/1000MG KBR 0-00-0	HU0482	
POLYETHYLENE FILM 0-00-0	HU0055	
POLYETHYLENE HIGH DENSITY	SP0049	
POLYETHYLENE OXIDIZED ACID NUMBER 15MG KOH/G/(KBR FILM)/SP-56	SP0056	
POLYETHYLENE, LD 0.015MM FILM 0-00-0	HU0708	
POLYETHYLENE, LINEAR FILM/XYL/ 0-00-0	HU0490	
POLYETHYLENE, OXIDIZED ACID NUMBER 15 MG KOH/G*	DA1168	
POLYOLEFIN [PP]	FB0111	Polypropylene
AMACO/OLEFIN(AMACO STAPLE) RED 19U POLYPROPYLENE NBS-E-0254	HU0942	
AMACO/OLEFIN/YELL TAPE POLYPROPYLENE 450-500 DEN NBS-H0258	DA3267	
HERCULON/POLYPROPYLENE/BROWN FIBER /STD491	HU0482	
CHEVRON CHEMICAL(VECTRA) / POLYLOOM(OLEFIN) GREEN NBS-K0033	DA3104	
PHILLIPS/OLEFIN(MARVESS) TOW 18.4U POLYPROPYLENE 3DEN NBS-F0249	DA3258	
CTA 87 A0436 POLYPROPYLENE	FI0065	
CTA 87 E0398 OLEFIN (POLYPROPYLENE)	FI0042	
CTA 87 E0469 POLYPROPYLENE	FI0085	
CTA 87 A0480 POLYPROPYLENE INTERMEDIATE BULK CONTAINER	FI0100	
POLYPROPYLENE ISOTACTIC/(KBR FILM)/SP-65	DA1172	
POLYPROPYLENE, ATACTIC 0.015MM FILM/TOL/ 0-00-0	HU0566	
POLYPROPYLENE, ATACTIC 0.02MM FILM 0-00-0	HU0943	
POLYPROPYLENE, ISOTACTIC	DA3322	
CTA 87 C0477 POLYPROPYLENE GEOTEXTILE FABRIC	FI0094	
POLYPROPYLENE, ISOTACTIC 0.025MM FILM 0-00-0	HU0942	
POLYPROPYLENE, SYNDIOTACTIC FILM 0-00-0*	HU0489	
POLY(STYRENE) FILM//KBR 0-00-0	HU0065	Polystyrene
POLY(STYRENE), ATACTIC FILM/CLF/CSI 0-00-0	HU0647	
MERRIFIELD POLYMER FLUKA 0.7M M. CL/G	F63871	
POLY(STYRENE:METHYLENE) FILM/CLF/CSI 0-00-0	HU0588	
F81413.SP F81413POLYSTYRENE STANDARD 300'000	PC2827	
F00926.SP F00926POLYSTYRENE STANDARD 400 CERTIFIED ACC. TO DIN	PC0050	
F00953.SP F00953POLYSTYRENE STANDARD 1'000'000 CERTI- FIED ACC. TO DI	PC0060	
COPOLYMER OF 96% STYRENE + 4% DIVINYL	F27822	
STYRENE/ALLYL ALCOHOL COPOLYMER 5.4%- 6.0% HYDROXYL*	SP0085	
STYRENE/DIENE COPOLYMER, OIL FREE	LU079B	
F00951.SP F00951POLYSTYRENE STANDARD 250'000 CERTIFIED ACC. TO DIN	PC0058	

Supplementary Table 3| Polymer types identified in the marine particle samples. Listed polymer types represent the accepted best hit scores (>0.7 out of 1) from the comparison of the measured spectra against the certified spectral database (S.T. Japan-Europe GmbH, Japan/Germany). For microplastic count and characterisation the individual polymer types where assigned into broader polymer groups (Methods). The spectra of the listed polymer types (except those labelled with asterisk) were used to examine the contamination level of the procedural blanks (Methods).

Fiji Image J command	Function
**run("8-bit");**	// converts image to 8-bit
**run("Set Scale...", "distance=2975 known=3000 pixel=1 unit=micron global");**	// scales the pixel number to micron units
**//run("Brightness/Contrast...");**<br><br>**resetMinAndMax();**	// resets brightness and contrast of an image
**run("Enhance Contrast...", "saturated=0.5");**	// saturates pixels
**run("Median...", "radius=2");**	// removes salt and pepper noise in 2 pixel neighbourhood (optional)
**run("Threshold...");**<br><br>**wait(1000);**<br><br>**setAutoThreshold("Default dark");**<br><br>**waitForUser("Threshold", "Please adjust threshold and then click OK");**	// enables manual setting of threshold brightness for every image
**run("Convert to Mask");**	// masks the background; converts image to black and white
**run("Fill Holes");**	// fills the background
**run("Labels...", "color=yellow font=14 show");**	// labels analysed particles in the output image
**run("Analyze Particles...",…);**	// measures horizontal dimensions and area of the particle and performs particle count.
**close();**	// End of macro

Supplementary Table 4| Fiji Image J macro for counting and characterising polymer-specific microplastics.

Supplementary References

1. Amigo, J. M., Babamoradi, H., Elcoroaristizabal, S. Hyperspectral image analysis. A tutorial. *Anal. Chim. Act.* **896:** 34-51 (2015).
2. Geladi, P., Grahn, H., Burger, J. Multivariate images, hyperspectral imaging: background and equipment. *Techniques and applications of hyperspectral image analysis* 1-15 (2007).
3. Karlsson, T. M., Grahn, H., van Bavel, B., Geladi, P. Hyperspectral Imaging and Data Analysis for Detecting and Determining Plastic Contamination in Seawater Filtrates. *J. Near Infrared Spec.* **24**(2)**:** 141-149 (2016).
4. Schindelin, J., et al. Fiji: an open-source platform for biological-image analysis. *Nat. meth.* **9**(7)**:** 676 (2012).
5. Renner, G., Schmidt, T. C., Schram, J. A New Chemometric Approach for Automatic Identification of Microplastics from Environmental Compartments Based on FT-IR Spectroscopy. *Anal. Chem.* **89**(22)**:** 12045-12053 (2017).
6. Vidal, M., Amigo, J. M. Pre-processing of hyperspectral images. Essential steps before image analysis. *Chemometr. Intell. Lab.* **117:** 138-148 (2012).
7. Bergmann, M. et al. High quantities of microplastic in Arctic deep-sea sediments from the HAUSGARTEN observatory. *Environ. sci. technol.* **51**(19)**:** 11000-11010 (2017).
8. Primpke, S., Lorenz, C., Rascher-Friesenhausen, R., Gerdts, G. An automated approach for microplastics analysis using focal plane array (FPA) FTIR microscopy and image analysis. *Anal. Meth.* **9**(9)**:** 1499-1511 (2017).
9. Simon, M., van Alst, N., Vollertsen, J. Quantification of microplastic mass and removal rates at wastewater treatment plants applying Focal Plane Array (FPA)-based Fourier Transform Infrared (FT-IR) imaging. *Water res.* **142:** 1-9 (2018).

10. Erni-Cassola, G., Gibson, M. I., Thompson, R. C., Christie-Oleza, J. A. Lost, but Found with Nile Red: A Novel Method for Detecting and Quantifying Small Microplastics (1 mm to 20 μm) in Environmental Samples. *Environ. sci. technol.* **51**(23)**:** 13641-13648 (2017).

11. Poulain, M. et al. Small Microplastics As a Main Contributor to Plastic Mass Balance in the North Atlantic Subtropical Gyre. *Environ. sci. technol.* **53**(3)**:** 1157-1164 (2018).

12. Jennings, B.R., Parslow, K., Ottewill, R. H. Particle size measurement: the equivalent spherical diameter. *P. Roy. Soc. Lon. A. Mat.* **419**(1856)**:** 137-149 (1988).

13. Kooi, M. et al. The effect of particle properties on the depth profile of buoyant plastics in the ocean. *Sci. Rep-UK* 6, 33882 (2016).

14. Yokota, K. et al. Finding the missing piece of the aquatic plastic pollution puzzle: Interaction between primary producers and microplastics. *Limnol. Oceanogr. Lett.* **2**(4)**:** 91-104 (2017).

15. Isobe, A., Iwasaki, S., Uchida, K., Tokai, T. Abundance of non-conservative microplastics in the upper ocean from 1957 to 2066. *Nat. commun.* **10**(1)**:** 417 (2019).

16. Cózar, A. et al. Plastic debris in the open ocean. *PNAS* **111**(28)**:** 10239-10244 (2014).

17. Pabortsava K. Abundance and size of microplastic particles in filtered seawater samples collected on a north-south transect in the Atlantic Ocean during cruise AMT26 (JR16001), September - November 2016. British Oceanographic Data Centre, National Oceanography Centre, NERC, UK. doi: 10.5285/aadd4168-0398-14f5-e053-17d1a68b059d (2020).

18. Geyer, R., Jambeck, J. R., Law, K. L. Production, use, and fate of all plastics ever made. *Science advances* **3**(7)**:** e1700782 (2017).

19. Jambeck, J. R. et al. Plastic waste inputs from land into the ocean. *Science* **347**(6223)**:** 768-771 (2015).

20. Lebreton, L.C. et al. River plastic emissions to the world’s oceans. *Nat. commun.* **8:** 15611 (2017).

21. Van Sebille, E. et al. A global inventory of small floating plastic debris. *Environ. res. lett.* **10**(12)**:** 124006 (2015).

22. Eriksen, M. et al. Plastic pollution in the world's oceans: more than 5 trillion plastic pieces weighing over 250,000 tons afloat at sea. *PloS one* **9**(12)**:** e111913 (2014).

23. Booth, A. M. et al. Microplastic in global and Norwegian marine environments: Distributions, degradation, mechanisms and transport.). SINTEF Oceans AS (2017).

24. Eakins, B. W. and G. F. Sharman, Volumes of the World's Oceans from ETOPO1, NOAA National Geophysical Data Center, Boulder, CO (2010).
