# Supplementary material for: High concentrations of plastic hidden beneath the surface of the Atlantic Ocean
Source: Nat Commun. 2020 Aug 18;11:4073. doi: 10.1038/s41467-020-17932-9 (PMC7434887; doi:10.1038/s41467-020-17932-9)
Supplement: Supplementary file 3 — Description of Additional Supplementary Files [file 41467_2020_17932_MOESM3_ESM.pdf]

Description of additional Supplementary files

File Name: Supplementary Data 1 (Microsoft Excel spreadsheet)

The file contains two sheets:

- Sheet ‘Number concentrations’ is a summary of data used to derive particle number concentrations (particles m^{-3}) of polymer-specific microplastics at each sampling location.
- Sheet ‘Mass concentrations’ compiles the data used for converting polymer-specific abundance from particle number concentrations units (particles m^{-3}) to mass concentration units (µg m^{-3}).

File Name: Supplementary Data 2 (Microsoft Excel spreadsheet)

Polymers/materials identified in the particle samples collected in the study region. The total number of polymer specific particles (N) in the 6 mm x 6 mm imaged area of each sample were determined only for Plastics/Resins and Rubbers material classes. Presence (marked as cross) and absence (marked as 0) of synthetic additives, cellulose derivatives and natural polymers in the samples are also included. Library hit score (range 0-1) indicates the spectral quality of each material detected. Number count for polymers with spectral quality below the accepted threshold (here, <0.7 and shown in bold italics) should be considered as bad/suspicious values.
