# Supplementary material for: High concentrations of plastic hidden beneath the surface of the Atlantic Ocean
Source: Nat Commun. 2020 Aug 18;11:4073. doi: 10.1038/s41467-020-17932-9 (PMC7434887; doi:10.1038/s41467-020-17932-9)
Supplement: Supplementary file 6 — Reporting Summary [file 41467_2020_17932_MOESM6_ESM.pdf]

nature research

Corresponding author(s): DPPR NCOMMS-19-3960434B

Last updated by author(s): Jul 22, 2020

Reporting Summary

Nature Research wishes to improve the reproducibility of the work that we publish. This form provides structure for consistency and transparency in reporting. For further information on Nature Research policies, see our Editorial Policies and the Editorial Policy Checklist.

Statistics

For all statistical analyses, confirm that the following items are present in the figure legend, table legend, main text, or Methods section.

n/a	Confirmed	
☐	☒	The exact sample size (*n*) for each experimental group/condition, given as a discrete number and unit of measurement
☐	☒	A statement on whether measurements were taken from distinct samples or whether the same sample was measured repeatedly
☐	☒	The statistical test(s) used AND whether they are one- or two-sided<br>*Only common tests should be described solely by name; describe more complex techniques in the Methods section.*
☐	☒	A description of all covariates tested
☐	☒	A description of any assumptions or corrections, such as tests of normality and adjustment for multiple comparisons
☐	☒	A full description of the statistical parameters including central tendency (e.g. means) or other basic estimates (e.g. regression coefficient) AND variation (e.g. standard deviation) or associated estimates of uncertainty (e.g. confidence intervals)
☐	☒	For null hypothesis testing, the test statistic (e.g. F, t, r) with confidence intervals, effect sizes, degrees of freedom and P value noted<br>*Give P values as exact values whenever suitable.*
☒	☐	For Bayesian analysis, information on the choice of priors and Markov chain Monte Carlo settings
☒	☐	For hierarchical and complex designs, identification of the appropriate level for tests and full reporting of outcomes
☒	☐	Estimates of effect sizes (e.g. Cohen's d, Pearson's r), indicating how they were calculated

Our web collection on statistics for biologists contains articles on many of the points above.

Software and code

Policy information about availability of computer code

Data collection	PerkinElmer Spectrum™ IMAGE (version R1.9.0.0030) and Spectrum™ 10 (version 10.5.4) software for Spotlight 400 (supplied with the instrument) were used to acquire hyperspectral IR images and to identify polymer type from individual infra-red spectra.
Data analysis	Spectra database from S.T. Japan-Europe GmbH, Germany/Japan (commercially available) was used as a reference polymer library to identify polymer types of the infra-red spectra collected.<br>FIJI Image J software (Java 8 version; open source; https://imagej.net/Fiji/Downloads) was used to for particle count and characterisation.<br>R (version 3.4.1; open source; https://www.r-project.org/) and Microsoft Office 2019 Excel (commercial) were used for statistical analysis and data visualisation.<br>Ocean Data Viewer (ODV; version 5.2.0 open source; https://odv.awi.de/software/download/) was used to plot the map of sampling locations and the section plot of isotherms in the study area.

For manuscripts utilizing custom algorithms or software that are central to the research but not yet described in published literature, software must be made available to editors and reviewers. We strongly encourage code deposition in a community repository (e.g. GitHub). See the Nature Research guidelines for submitting code & software for further information.

Data

Policy information about availability of data

All manuscripts must include a data availability statement. This statement should provide the following information, where applicable:

- Accession codes, unique identifiers, or web links for publicly available datasets
- A list of figures that have associated raw data
- A description of any restrictions on data availability

All polymer-specific data underlying this study can be downloaded from the British Oceanographic Data Centre (BODC; doi: 10.5285/aadd4168-0398-14f5-e053-17d1a68b059d) or are available from the corresponding author upon request. The Argo data of monthly mixed layer climatology under product name 'May 2018 netcdf format' were downloaded from the Argo Mixed Layer website (http://mixedlayer.ucsd.edu/) hosted by Scripps Institution of Oceanography, UC San Diego. The Argo data used to create this climatology were collected and made freely available by the International Argo Program and the national programs that contribute to it (http://www.argo.ucsd.edu, http://argo.jcommops.org). The Argo Program is part of the Global Ocean Observing System (doi.org/10.17882/42182#56126). The CTD profile data collected during the AMT26 JR16001 expedition were provided by and are freely available from BODC (doi: 10.5285/aa51baf6-2095-6c28-e053-6c86abc0d7f7).

Field-specific reporting

Please select the one below that is the best fit for your research. If you are not sure, read the appropriate sections before making your selection.

☐ Life sciences ☐ Behavioural & social sciences ☒ Ecological, evolutionary & environmental sciences

For a reference copy of the document with all sections, see nature.com/documents/nr-reporting-summary-flat.pdf

Ecological, evolutionary & environmental sciences study design

All studies must disclose on these points even when the disclosure is negative.

Study description	This is a field study of small plastic debris termed 'microplastics' collected at twelve stations on a meridional transect in the Atlantic Ocean via in situ filtration at three discrete depths (10 to 270 m below the sea surface). Microplastics measurements in all 35 samples (no surface sample at station 01) were conducted using Fourier-Transform Infrared (FTIR) spectroscopic imaging down to a resolution of 25 micron pixels and with a specific focus on detection of three most littered plastics (polyethylene, polypropylene and polystyrene). The polymer-specific number concentrations were converted to mass concentrations based on the measured particle size in two dimensions and the assumption of certain 3D shapes. Different particle shapes were tested to determine minimum and maximum values for this conversion. The observed polymer-specific weights were averaged over the investigated depth horizon and scaled up to the width of the Atlantic basin. The magnitude of pollution with these three polymers in the upper 200 m was discussed in a context of previously-estimated load of ocean plastics and input of these contaminants to the ocean over the past 65 years.
Research sample	Each sample (total of 35) is an aliquot of the filtered marine particles including plastic debris of > 25 micron in size collected from three selected water depths in the Atlantic Ocean. These samples are meant to represent the extent of contamination of the Atlantic sea-surface and interior with the most littered plastic material.
Sampling strategy	The sampling for microplastics was conducted alongside the Atlantic Meridional Transect research cruise AMT 26 (~ 46°N – 53°S) in September-November 2016 as an additional research effort to the core observations of this annually repeated oceanographic field survey. The cruise track and the location of sampling stations including those for microplastics were thus determined in accordance with the main research targets of the AMT programme (https://www.amt-uk.org/).<br><br>Sampling strategy involved filtering large volumes of seawater at three selected depths (surface, intermediate, and mesopelagic) based on hydrographic settings (specifically, depth of the mixed layer). Smaller aliquots were taken from these samples and after removal of organic material, the chemical composition of the filtered particle mixture was measured using Fourier-Transform infrared (FTIR) spectroscopic imaging down to resolution of 25 microns. The study focussed on the concentrations of three, commercial most prominent polymer types of polyethylene, polypropylene and polystyrene.<br><br>Contamination prevention and control were essential part of the sampling and analysis strategy. All steps involving sample collection, processing and analysis were performed in the air-controlled environment and using clean and where possible non-plastic laboratory-ware. To assess the contamination bias, procedural blanks (in triplicates) were prepared on board of the ship, then processed and analysed in exactly the same way as the samples.<br><br>No statistical sample size calculation was performed, these samples are sufficient/representative because they i) originate from large volumes of seawater (> 500 L), are ii) contamination-free, and iii) contain sufficient number of polymer-specific plastic per imaged area.
Data collection	Data collection and logging was performed by the corresponding author and involved the following procedure:<br><br>i) Hyperspectral infrared (IR) images of the filtered particle samples were acquired using FTIR spectroscopic imaging in transmission mode over spectral range of 4000–750 cm-1 at 8 cm-1 spectral resolution and 25 micron pixel resolution applying 4 co-added scans.<br>ii) Chemical composition of the imaged particle sample was explored using chemometric technique of principal component analysis (PCA)<br>iii) Multiple spectra were collected from each principal score and their chemical composition (here, polymer type) were determined

through comparison against the spectra in the reference polymer database.
iv) Good quality spectra corresponding to the polymer types of interest were selected and used as reference spectra to reconstruct the original infrared image into a correlation map, in which correlation coefficient between that polymer-specific reference spectrum and every pixel/spectrum on the original IR image would show the locations of and areas occupied by that specific polymer type.
v) Polymer-specific images (correlation maps) were imported into FIJI-Image J software for particle analysis (particle number count) and characterisation (measurements of area, horizontal dimensions and shapes).

In addition to particle number count, the polymer-specific mass concentrations were calculated based on particle size and shape, with different tests (here, assumptions of particle shape) conducted to determine minimum and maximum values for this type of conversion.

Timing and spatial scale

Sample collection at sea: the samples were collected aboard RRS James Clack Ross during the passage of the Atlantic Meridional Transect cruise AMT 26 (JR16001). The expedition departed Immingham, UK on the 20th September 2016 and arrived in the Falkland Islands, UK on the 3rd November 2016 after travelling 7829 nautical miles (~14500 km from 45.7°N 13.6 °W to 53.4°S 41.5°W) via the Azores and South Georgia.

FTIR Imaging: the collection of hyperspectral infrared images of all 35 samples and procedural blanks were completed in a period from 30.05.2018 to 30.07.2018. Image processing and particle analysis were completed by December 2018.

Data exclusions

The identification of chemical composition/polymer type of a particle was deemed not successful and not considered in this study when the hit quality between the measured and the reference library spectrum of that particle was below 0.7 (scale 0-1). This criteria is equivalent to the quality score threshold reported/recommended in previous studies (e.g. Primpke et al. 2017, Bergmann et al. 2018).

Note that the data are not available for station 01 at 10 m depth due to the pump failure.

Reproducibility

Sampling: Due to logistical and time constrains, water samples for microplastics were collected only once at a given location/depth.

FTIR Imaging: for each sample, four square selections of equal area termed ‘markers’ were imaged at 4 scans per pixel to enhance spectral quality. These four markers were treated as replicated to account for the uneven distribution of plastic particles on the sample filter (see similar approach in Peeken et al. 2017). We calculated the mean and standard deviation of the four replicates to determine particle count of the total imaged area of a sample and then used these values to extrapolate to the total area filtered.

Randomization

Samples were grouped based on sampling location (station ID) and depth (surface, intermediate, mesopelagic).

Spectra of different types/library IDs of polyethylene (e.g. linear, low density, high density), polypropylene (atactic, isotactic) and polystyrene (e.g. film, standard) were generalised into broader polymer groups.

Blinding

Our study is based on observational/field data collected during the annually repeated oceanographic field survey JR16001 (AMT 26) Atlantic Meridional Transect programme (https://www.amt-uk.org/) at locations determined in accordance with its main research targets. Our study does not include designed laboratory trials or experiments and thus blinding is not relevant to our study.
We followed published/approved techniques and applied recommended procedural precautions to minimise any bias of our results. Specifically, all samples were collected a consistent manner at all locations and analysed using the same analytical approach and equipment. The limitations associated with sampling and analysis were appropriately acknowledged in the relevant sections of the manuscript. We note that introducing microplastic particles from the surrounding, laboratory items and equipment into the sample could bias the results. In the present study, the rigorous contamination prevention measures were taken and included work in the clean, air-quality controlled environment (e.g. laminar flow cabinet, ISO-5 clean laboratory, covered microscope imaging unit) and using cleaned and where possible non-plastic laboratory-ware. The procedural blanks (triplicates) were taken on board of the ship and processed exactly as samples to account for any contamination associated with sample collection, handling and analysis.
Data analysis and statistical comparisons were done with respect to sample location in the Atlantic and/or in the water column, polymer type and particle size of microplastics debris. Limitations of comparison of our results to data published in literature (e.g. different sampling and analytical techniques used) were duly acknowledged in the manuscript.

Did the study involve field work? ☒ Yes ☐ No

Field work, collection and transport

Field conditions

Water column samples were collected using in situ pumps aboard RRS James Clack Ross during the passage of the Atlantic Meridional Transect cruise AMT 26 in September-November 2016. The shallowest sampling depth was always at 10 m below the surface to obtain samples representative of the upper water column. We collected microplastics at two depths below the base of mixed layer to measure their dispersal into the ocean interior. The depth of the mixed layer was determined from the conductivity-temperature-depth (CTD) profiles collected prior to each deployment of the pumps and employing a fixed temperature-based criterion described in the methods section of the manuscript. The relevant CTD data are available on request from British Oceanographic Data Centre (https://www.bodc.ac.uk/projects/data_management/uk/amt/). Additional information on field conditions during the expedition can be found in the AMT 26 cruise report available at https://www.amt-uk.org/Cruises/AMT26.

Location

Sampling dates and geographical locations of stations sampled for microplastics are listed below:

Station 01: sampled on 25.09.2016 at 45.8°N 13.6 °W; sampled depths were 10 m (pump failure), 60 m, 100 m

	Station 03: sampled on 29.09.2016 at 37.7°N 24.9°W; sampled depths were 10 m, 65 m, 150 m<br><br>Station 06: sampled on 04.10.2016 at 21.2°N 29.7°W; sampled depths were 10 m, 100 m, 200 m<br><br>Station 08: sampled on 06.10.2016 at 14.5°N 28.6°W; sampled depths were 10 m, 50 m, 150 m<br><br>Station 09: sampled on 07.10.2016 at 11.2°N 27.7°W; sampled depths were 10 m, 50 m, 150 m<br><br>Station 12: sampled on 10.10.2016 at 1.2°N 25.3°W; sampled depths were 10 m, 70 m, 170 m<br><br>Station 15: sampled on 13.10.2016 at -9.1°N 25°W; sampled depths were 10 m, 100 m, 200 m<br><br>Station 18: sampled on 17.10.2016 at -21.3°N 25°W; sampled depths were 10 m, 170 m, 270 m<br><br>Station 19: sampled on 18.10.2016 at -24.5°N 25°W; sampled depths were 10 m, 140 m, 240 m<br><br>Station 23: sampled on 22.10.2016 at -37.3°N 28.4°W; sampled depths were 10 m, 100 m, 200 m<br><br>Station 27: samples on 27.20.2016 at -50.3°N 34.1°W; sampled depths were 10 m, 100 m, 200 m<br><br>Station 30: sampled on 31.10.2016 at -53.4°N 41.5°W; sampled depths were 10 m, 100 m, 200 m
Access & import/export	All microplastics samples were collected in the international waters, and hence no diplomatic clearance for sampling was required. No import permit was required for the transport of the filtered microplastics samples as filtered seawater is not controlled by import licensing regulations (per communication with the Animal and Plant Health Agency (APHA); contact Imports@apha.gsi.gov.uk; email communication with the agency prior to the cruise can be provided upon request).
Disturbance	Collecting seawater samples for microplastics did not disturb the open ocean habitat in any significant way. No vulnerable habitats were sampled in this study.

Reporting for specific materials, systems and methods

We require information from authors about some types of materials, experimental systems and methods used in many studies. Here, indicate whether each material, system or method listed is relevant to your study. If you are not sure if a list item applies to your research, read the appropriate section before selecting a response.

Materials & experimental systems

n/a	Involved in the study
☒	☐ Antibodies
☒	☐ Eukaryotic cell lines
☒	☐ Palaeontology and archaeology
☒	☐ Animals and other organisms
☒	☐ Human research participants
☒	☐ Clinical data
☒	☐ Dual use research of concern

Methods

n/a	Involved in the study
☒	☐ ChIP-seq
☒	☐ Flow cytometry
☒	☐ MRI-based neuroimaging
