# Supplementary material for: High concentrations of plastic hidden beneath the surface of the Atlantic Ocean
Source: Nat Commun. 2020 Aug 18;11:4073. doi: 10.1038/s41467-020-17932-9 (PMC7434887; doi:10.1038/s41467-020-17932-9)
Supplement: Supplementary file 2 — Peer Review File [file 41467_2020_17932_MOESM2_ESM.docx]

Peer Review Information

**Journal:** Nature Communications

**Manuscript title:** Click here to enter text.
High concentrations of plastic hidden beneath the surface of the Atlantic Ocean

**Corresponding author name(s):** Dr Katsiaryna Pabortsava

**Editorial notes:**

**none**

**Reviewer comments & decisions:**

**Reviewer comments, first version:**

Reviewers' comments:

Reviewer #1 (Remarks to the Author):

In this paper authors have sampled the upper 200m of the Atlantic Ocean and have detected a major sink of PE, PP and PS in the upper layers of this water body. Authors go further and do a calculation/extrapolation to the entire plastic budget and find that a major fraction of the plastic in the oceans was detected. While extremely timely, interesting and filling a current sampling gap, I consider the data has to be taken with caution and analysed more carefully in order to make such large extrapolations, which doesn’t mean conclusions are not valid.

Major comments:
- I don’t understand why all polymer types were not reported. Authors seem to have this information but they didn’t include it. Authors need to add all polymer types as this will give a broader view of the pollution problem magnitude and support that PE, PP and PS are really the most prevalent polymers in this part of the ocean basin. The additional data will make the data (which is currently very variable) much more trustworthy.
- Authors have collected particles down to 1um in size but only report the >25um fraction (hence, the smallest particle detected was of >30um). The text is written eluding the smaller fraction and not explaining why this fraction was excluded… it’s not until I read the M&M section that I realised WHY they did NOT detect particles smaller than 30um. Authors need to supply the data from the smaller fraction as, currently, this missing data makes the results presented very weak. Including this information would largely increase the impact of this paper as it will explain the fate of ALL smaller plastics.
- When extrapolating the data, vertical as well as horizontal variability needs to be taken into account to obtain a much more robust plastic budget which seems to be the main message of the paper. Authors also elude the fact their hotspots of plastic abundance does not correlate with previous data (in the gyres) and the excessively high variability in the data.

Minor comments:
ABSTRACT.
The entire abstract needs rewriting… currently it is not clear. For example:
Line 10. Remove ‘massive’ as this is a subjective term.
Lines 10-11. This sentence doesn’t seem right… very ambiguous and not at all clear. What plastic types? What sizes? …
Line 11-13. If the previous sentence is true, why should there be other polymer types and plastics in the sediment?
Lines 15-18. This sentence should the intro to the abstract, why does it come after the final conclusion of the study?

INTRODUCTION
Nice introduction that highlights the knowledge gap.
Line 55. Please stop using ‘massive’ so freely. It’s a 10,000 km transect, not a ‘massive’ 10,000 km transect. Why over exaggerate a sentence that is so concise?
Line 56. What ‘key polymers’?
Line 56. “… down to 25 um in size…”

RESULTS
Fig 1b. Change the variable ‘Pressure’ for Depth as this will give a better idea of the location within the water column that was sampled.
Lines 72-76. The detail of the depths at which the SAPs were deployed is over-complicated. Rephrase to simplify. This could be achieved by addressing my previous comment for fig 1b.
Line 97. 200m is not the deep ocean.
Fig 2. It is strange that there is no peak of plastic concentrations in the gyres, where it is well established that plastics converge in the so-called ‘garbage patches’.
Line 114 and Fig 3. This is very puzzling and a major concern. Researchers filtered seawater through 1 and 55um filters but their detection limit was only 32um in size. What does this mean? Where is the limiting element in the protocol that doesn’t allow the detection of smaller particles? Or authors suggest they aren’t there? In Fig 3 authors shouldn’t put 0 as their smallest size. Change for 32um. In fact, did authors try to represent in a log scale to make the size distribution more obvious?
Not trying to be mean with this comment, but the enormous variability of plastics found makes me wonder how trustworthy this dataset is. For example, is the strange size/weight distribution of PP with depth (i.e. mesopelagic, and seen both in Figures 1 and 3) due to maybe just 3 very large particles? How would the final conclusions change with the random addition of one large particle of a polymer type? The sample number may need to be higher. Maybe by imaging the entire filter?

DISCUSSION
Overall, the discussion is excessively long and too speculative with a highly variable dataset that doesn’t support most of the comments, or being so variable, it’s not even worth discussing. Drastically reduce this section to the robustly findings supported by the data. For example: all discussion about PS (its detection is too variable, no need to speculate).
Also reduce repetitiveness. For example: lines 150-161 overlaps with discussion in section line 188.
Line 171. EPS has so little density that it wouldn’t even enter the water. They are usually just blown to the coast.
Line 194. Microscopy or spectroscopy?
Lines 188-214. While comparing their concentrations with other studies, authors need to discuss their results in terms of location. The most striking finding is that maximum densities are NOT found in the gyres, a well-established hotspot in previous studies.
Lines 265-272. This back-of-the-envelope calculation, and which is the major conclusion of this study, need a bit more accurate estimation. Not taking the average of all vertical and horizontal distribution, but modelling these distributions and doing a more accurate calculation of the total budget.

M&Ms
The methods description is very thorough and sometimes difficult to follow… From what I understand, SAPs collected the particles on 55 and 1 um filters that were then rinsed off with ASW. Were both filters mixed? Do authors have what was collected on the 55um and 1um filters separately?
Lines 383-385. This data needs to be included to have a full picture of the total budget. Now I understand why the smallest particle found was of over 30um… why wasn’t the silver 0.8um filters analysed and reported here?
Line 386. I am not aware of the exact methodology of FTIR imaging and, hence, can’t revise this section correctly. But it would be nice to have a better explanation of all the 6 x 6 mm2 and 3 x 3 mm2 and sections, etc. It’s a bit confusing what was imaged and how the data was extrapolated (line 425). Couldn’t the whole filter be imaged?


Reviewer #2 (Remarks to the Author):

1. Comments for Author
In this paper Pabortsava1 & Lampitt conducted during the 26th ATM cruise microplastic (MP) sampling on a latitudinal transect across the Atlantic (~ 46°N – 53°S) in September to November 2016. They filtered large volumes of water at three-selected depth (surface, intermediate, mesopelagic) based on hydrographic settings. From the samples, small aliquots were taken and after removal of organic material, the filters were subjected to MP measurements using Fourier-Transform infrared (FTIR) imaging down to a resolution of 25 µm pixels. The study focussed on the three, commercial most prominent polymer types of polyethylene (PE), polypropylene (PP) and polystyrene (PS). Beside particle number they also provide the polymer-specific mass concentrations, which was calculated based on particle size and shape, including different test to have minimum and maximum values for this type of conversion.
The average observed polymer weight from the various ocean depth were further basin wide upscaled and discussed, how MP in the size range of 32-651 μm will contribute to the overall plastic pollution budgets, previously estimated. The authors claim that with their study they can show that there is not a missing sink for plastic in the ocean, but rather, even when focussing only on three compounds (PE, PP &PS) and the small particles, they can already account for all the cumulative plastic supply reaching the Atlantic Ocean since the early 50ties. Thus, they consider that the overall input of plastic litter into the ocean must be much larger then hitherto accounted for. However, this conclusion is based on a budget, which is not properly presented in the manuscript and will need a revision, before the paper can be accepted.
The role of plastic litter in the ocean is of major concern with high societal relevance. The authors present the first measurements focussing on small particles of 32-651 μm, on an ocean wide transect covering different geographic regions in oceanography and distinct depth horizons and thus shed new light on the overall plastic pollution in a global context. However, these are only 12 stations and the question remains, how representative these sample collections are for the chosen transect. Given the long history of AMT cruises, the authors should discuss this in a broader context.
Overall, sampling and measurements appear to be of high quality and the provided contamination measures seem to be appropriate for this type of analysis. The only unclear part of the investigation is the use of a Folsom splitter and the resulting effect on the overall numbers derived in this study. In addition, parts of the discussion are inconsistent and need some revision.
Overall, I assume that this study will rock the field of microplastic research, since it again puts the attention to the very small MP, which are not captured with common methods as manta trawls, which are usually recommended to use for monitoring of surface sea water (e.g. GESAMP (2019). Guidelines or the monitoring and assessment of plastic litter and microplastics in the ocean). This type of sampling devices only capture particles larger than 300µm, which according to this study are of minor importance in the overall context of plastic weight in the ocean. If they can come up with a convincing basin-scale budget, it will also stimulate the discussion about re-evaluation of plastic sources for the marine environment. Statistical analysis have been performed adequately and the overall detailed description of sample handling and identification procedure seem to be appropriate, thus other scientists in the field should be able to replicate this type of MP measurements.


Please find below more specific points, which need some attention before the publication can be accepted:
Abstract:
Line 9: It need to be clarified, how the basin-scale numbers were obtained. (see again below)
Results:
Line 71: employing a fixed temperature-based criterion of ref24. Please explain in more detail which MLD criteria has been used
Line 75: also refer to Supplementary Table S1
Line 81: in all procedural blank. Please explain better and refer to the details in the supplements
Discussion:
Overall, the discussion is not consistent as to why certain types of polymers float or sink and requires clear lines of argumentation in the revised form of the manuscript. In addition, the authors should put more attention to the fact, how reliable their basin-scale estimates are.
Line 159ff: Please give references to this statement, what specific gear is used. Other studies in the Southern Ocean also rather find PA in their samples, which is usually attributed to fishing gear.
Line 169 ff: Sentence seemed to be out of context and wrong since PE and PS are typical lighter compared to seawater, as the authors state in their own supplements Line 622 ff ((PE=0.880-0.960 g cm-3; PP=0.895-0.92 g cm-3; PS=0.960–1.04 g cm-3)
Line 181: “To address…..” Please specify in more detail
Line 184-185: Please give reference
Line 202: Incorrect, numbers in sea ice were orders of magnitude higher
Line 206: How do data compare to the North Atlantic (e.g. Lusher et al. 2015)
Line 226: “short residence time of…” This seems not so clear from the data presented in Fig 4, which show only for PP the short residence time in the surface, while PE and PS decrease with depth.
Line 227ff: Line of arguments thus not clear
Line 260ff: How were the estimates done; maybe this section is missing in the result part?
Line 266: What is the whole area?
Line 274: 6%, how is this number derived?
Line 302: Arctic not Antarctic

Methods:
Line 353: “thoroughly cleaned prior to every” How were they cleaned?
Line 374ff: If you split 1000ml you should receive 250ml. How was the next step of aliquots done, since only 100ml were used for further treatment of the samples? Which effect has the Folsom splitter on MP contamination and did you test, how this affects your overall calculation of concentrations?

Data availability:
Line 462: For publication in Nature Communications I think it is required to upload the data in a public data base.

In conclusion, I think this paper is highly valuable to be published in Nature Communication however; I think the authors need to clarify the budget section of the paper and fix a few more inconsistencies before the paper can finally be accepted.

**Author rebuttal, first version:**

**GENERAL RESPONSE TO THE REVIEWERS**

We are very grateful to receive a constructive review of our manuscript entitled “**High level plastic contamination in the Atlantic interior**”. We also appreciate the time and effort you took to provide such insightful guidance. We carefully considered your comments and recommendation and applied them to improve our manuscript.

Based on your collective input, our revised manuscript now contains:

- Clarified data sources and methodology, including the use of Folsom splitter and calculation of the plastic budgets in the Atlantic
- Improved discussion of the results in the context of previous observations and model-based predictions.
- Improved Figures 1 and 3
- Additional supplementary data to support the findings and assumptions for the plastic budget calculations.

We hope that these revisions improved the manuscript such that it is now accepted for publication in *Nature Communications*.

**RESPONSE TO REVIEWER #1’S COMMENTS**

Reviewer #1 (Remarks to the Author):

**Comment 1:** In this paper authors have sampled the upper 200 m of the Atlantic Ocean and have detected a major sink of PE, PP and PS in the upper layers of this water body. Authors go further and do a calculation/extrapolation to the entire plastic budget and find that a major fraction of the plastic in the oceans was detected. While extremely timely, interesting and filling a current sampling gap, I consider the data has to be taken with caution and analysed more carefully in order to make such large extrapolations, which doesn’t mean conclusions are not valid.

**Response 1:** *We thank Reviewer #1 for the feedback on our manuscript. Below we provide the response to each of Reviewer #1’s comments and recommendations.*

Major comments:

**Comment 2:** I don’t understand why all polymer types were not reported. Authors seem to have this information but they didn’t include it.

***Response 2:*** *We have excluded data on polymers for which the analytical confidence is poor or where their abundance is extremely low. As with all analytical methods, there are limitations with the ones used in our study. For example, not all polymers are equally resistant to the extraction solution used (Dehaut et al. 2016, Kuhn et al. 2017). Although FTIR imaging is recognised as one of the most powerful and widely available techniques for relatively quick and reliable detection of synthetic polymers, not all plastics will have equally high sensitivity when scanned in FTIR transmission mode (e.g. Loeder et al. 2015, Elert et al. 2017). We thus do not report the results which we know could potentially be the analytical artefacts and would lead to erroneous conclusions about the abundance of these contaminants in the marine environment. We would of course prefer to be able to obtain high-quality data on all synthetic polymers from a single sample and using a single, fast and efficient approach. This is however currently beyond the analytical capability of any research lab. The conclusions of our study are however robust and the limited number of polymer groups reported do not in any significant way detract from this.*

**Comment 3:** Authors need to add all polymer types as this will give a broader view of the pollution problem magnitude and support that PE, PP and PS are really the most prevalent polymers in this part of the ocean basin. The additional data will make the data (which is currently very variable) much more trustworthy.

***Response 3:*** *As commented above, there are methodological challenges, as with any analyses, which preclude us from reporting with confidence all polymer types detected in our samples. This does not however influence the significance of our observations in the context of the global marine plastic contamination and does not detract from the major conclusions of our study.*

*Our study does not intend to demonstrate that PE, PP and PS are really the most prevalent polymers in the Atlantic, but to determine their abundance and distribution in the basin. We chose to focus our study on the polymer groups which i) together account for more than half of plastic litter generated globally (Geyer et al. 2017), ii) were frequently reported in the earlier surveys of marine plastic contamination (Erni Cassola et al. 2019 and references therein), and iii) are of high analytical confidence. Spatially and temporally extensive and internally consistent observations of polymer-specific plastics in the ocean are required to make strong conclusions on the prevalence of specific plastic types in the marine environment. Our study, which availed the sampling opportunity of the annually repeated Atlantic Meridional Transect programme (*[*https://www.amt-uk.org/*](https://www.amt-uk.org/)*), sets an example for how such observations can be conducted in the future.*

**Comment 4:** Authors have collected particles down to 1um in size but only report the >25um fraction (hence, the smallest particle detected was of >30um). The text is written eluding the smaller fraction and not explaining why this fraction was excluded… it’s not until I read the M&M section that I realised WHY they did NOT detect particles smaller than 30um.

***Response 4:*** *We do not report microplastic particles in size range 1-25 µm because we currently have a lower confidence in that dataset. This specifically concerns noisier IR spectra obtained for higher resolution measurements in reflectance mode and as a result lower quality of the comparison against the spectra in polymer library. These measurements are also more time-intensive, and thus much smaller areas of the filter were analysed, which introduces additional uncertainty when extrapolating these data. In Methods section, we refer to 1-25 µm size fraction of our samples to ensure that the procedure we followed could be reproduced in future studies (lines 424-431).*

**Comment 5:** Authors need to supply the data from the smaller fraction as, currently, this missing data makes the results presented very weak. Including this information would largely increase the impact of this paper as it will explain the fate of ALL smaller plastics.

***Response 5:*** *As stated in the response above, we had a lower confidence in the data on 1-25 µm microplastics and thus they were excluded from the reported data. We presented the size fraction which is well above the detection limit of the FTIR imaging techniques (~ 3 µm) as these data are of our highest confidence.*

*The reported size range of microplastics by no means detracts the unprecedented scope and importance of our observations for understanding the marine plastic contamination of the ocean on basin- and global scales. It also does not diminish the significance and implications of our conclusions about the relatively minor importance of commonly sampled large plastic debris (> 250 µm) in the overall context of plastic weight in the ocean. It would undoubtedly be useful to constrain the abundance of 1-25 µm size category of microplastics, but to explain the fate of ALL smaller plastics, the debris in nano-size range (<1µm) should also be included, an aspiration which currently poses a huge sampling and analytical challenge for any research group.*

**Comment 6:** When extrapolating the data, vertical as well as horizontal variability needs to be taken into account to obtain a much more robust plastic budget which seems to be the main message of the paper.

***Response 6:*** *Spatial variability in environmental sampling is often large and, in some cases, changes to a current understanding are hard to support. This is most definitely not the case for our data which radically change the accepted viewpoint. Although spatial variability is large, this does not detract from the significance of the conclusions. We would of course prefer to have 10 times the amount of data and to have these data for every month of the year and with an explanation for the variability. This is beyond current sampling or analytical capability of any research group. The conclusions are however robust and the uncertainties and variability do not in any significant way detract from this.*

**Comment 7:** Authors also **elude** the fact their hotspots of plastic abundance does not correlate with previous data (in the gyres) and the excessively high variability in the data

***Response 7:*** *In the revised manuscript we discussed the observed distribution of microplastics in the surface and subsurface waters of the Atlantic and specifically in relation to the identified accumulation hotspots of floating plastic debris in the subtropical gyres (lines 180-214). We also highlighted the extremely high spatial variability of plastic abundance reported in earlier surveys in the Atlantic gyres (Law et al. 2010, Eriksen et al. 2014, Cozar et al. 2014, Enders et al. 2015; lines 192-194).*

Minor comments:

ABSTRACT.

**Comment 8:** The entire abstract needs rewriting… currently it is not clear.

***Response 8:*** *We incorporated the reviewer’s suggestions in the revised abstract.*

For example:

**Comment 9:** Line 10. Remove ‘massive’ as this is a subjective term.

**Response 9:** *Done*

**Comment 10:** Lines 10-11. This sentence doesn’t seem right… very ambiguous and not at all clear. What plastic types? What sizes? …

***Response 10****: this sentence has been revised (lines 9-11).*

**Comment** **11**: Line 11-13. If the previous sentence is true, why should there be other polymer types and plastics in the sediment?

***Response 11:*** *Our study demonstrates that accounting for the >25 µm size category of the three most discarded polymer types in the top 5.3% of the Atlantic Ocean balances the amount of plastic litter that is predicted to have entered the basin over the past 65 years. We however also conclude that there is no missing sink of ocean plastic, and, on the contrary, the plastic inputs into the ocean over the past 65 years have been grossly underestimated. The following arguments support our conclusions:*

i) *PE, PP and PS polymers represent 56% of plastic waste generated globally (Geyer et al. 2017 and lines 50-512) and plastics of other polymer types have been detected previously in the surface ocean (e.g. Enders et al. 2015, Kanhai et al. 2017) and in sediments (e.g. Bergman et al. 2017).*
ii) *The particle size detection limit in this study is 25 µm. Earlier studies report the presence of microplastics down to 10 µm in size floating in the surface ocean (Enders et al. 2015) and deposited/buried on the seabed* *(Bergman et al. 2017). Contamination with plastic debris in nanoplastics is of increasing concern (Mitrano 2019), yet unquantified.*
iii) *Deep-sea sediments are considered to be the ultimate and likely the major sink for plastic litter (Booth et al. 2017, Wright and Kelly 2017). They are however severely undersampled on spatial and temporal scales and also with respect to small microplastics. Bergmann et al. 2017 demonstrated large amounts of microplastic >11 µm is size buried on the Arctic seafloor. It is thus reasonable to assume that a significant portion of plastic litter would be found below the depths sampled in our study and in transition to the seabed.*

**Comment 12:** Lines 15-18. This sentence should the intro to the abstract, why does it come after the final conclusion of the study?

***Response 12:*** *This sentence coveys the conclusions and implications of our observations and hence comes in the final part of the abstract. Note that the abstract was revised based on the suggestions of both reviewers.*

INTRODUCTION

**Comment 13:** Nice introduction that highlights the knowledge gap.

***Response 13:*** *we thank the reviewer for this positive feedback*

**Comment 14:** Line 55. Please stop using ‘massive’ so freely. It’s a 10,000 km transect, not a ‘massive’ 10,000 km transect. Why over exaggerate a sentence that is so concise?

***Response 14:*** *Done*

**Comment 15:** Line 56. What ‘key polymers’?

***Response 15:*** *This sentence was clarified (lines 48-49 and 54-55)*

**Comment 16:** Line 56. “… down to 25 um in size…”

***Response 16:*** *Corrected. We thank the reviewer for spotting this.*

RESULTS

**Comment 17:** Fig 1b. Change the variable ‘Pressure’ for Depth as this will give a better idea of the location within the water column that was sampled.

***Response 17:*** *We thank the reviewer for this suggestion. The title of the y-axis in Fig. 1b now reads ‘Depth (m).*

**Comment 18:** Lines 72-76. The detail of the depths at which the SAPs were deployed is over-complicated. Rephrase to simplify. This could be achieved by addressing my previous comment for fig 1b.

***Response 18:*** *The description of sample collection with SAPs was clarified and incorporates the suggestions of both reviewers (lines 62-76).*

**Comment 19:** Line 97. 200m is not the deep ocean.

***Response 19:*** *We corrected this to ‘...polymer-specific plastic penetrating deeper into the ocean interior’ (line 97).*

**Comment 20:** Fig 2. It is strange that there is no peak of plastic concentrations in the gyres, where it is well established that plastics converge in the so-called ‘garbage patches’.

***Response 20:*** *The reviewer raises an important point which we addressed in the revised manuscript (lines 180-214); also see the response to Comment 7).*

**Comment 21:** Line 114 and Fig 3. This is very puzzling and a major concern. Researchers filtered seawater through 1 and 55um filters but their detection limit was only 32um in size. What does this mean? Where is the limiting element in the protocol that doesn’t allow the detection of smaller particles? Or authors suggest they aren’t there?

***Response 21:*** *We apologise if the method of sampling processing and analysis was not immediately clear to the reviewer. One of the reasons for collecting a size fractionated marine particle sample is to avoid clogging of the filters, especially when SAPs are deployed in the surface waters and/or in the areas where the productivity is high. For microplastics analysis, samples were prepared (pre-filtered) to best utilise the capabilities of the FTIR imaging system to image particles at multiple resolutions, including 25 µm. Hence, our choice of the mesh size for pre-concentrating microplastics post-digestion was stainless steel mesh with the aperture of 25 µm.*

**Comment 22:** In Fig 3 authors shouldn’t put 0 as their smallest size. Change for 32um. In fact, did authors try to represent in a log scale to make the size distribution more obvious?

***Response 22:*** *We thank the reviewer #1 for the suggestion. We modified the figure to indicate the lowest size of microplastics detected in our samples (32 µm). We however maintained the linear scale of the x-axis to compare the shapes of the distributions (per individual plastic type and sampling depth) irrespective of the number of data points making up each distribution (see legend of Figure 3, lines 541-576).*

**Comment 23:** Not trying to be mean with this comment, but the enormous variability of plastics found makes me wonder how trustworthy this dataset is.

***Response 23:*** *As commented above, there is certainly spatial variability in our dataset as with all environmental data and we do not have robust explanations for this. This does not however influence the major conclusion of our study.*

**Comment 24:** For example, is the strange size/weight distribution of PP with depth (i.e. mesopelagic, and seen both in Figures 1 and 3) due to maybe just 3 very large particles? How would the final conclusions change with the random addition of one large particle of a polymer type? The sample number may need to be higher. Maybe by imaging the entire filter?

***Response 24:*** *In the revised manuscript, for each polymer group we provided the absolute count of microplastics detected in the total 6×6 mm^2^ area imaged (Supplementary Table S1). The elevated weight concentrations of PP observed in the mesopelagic layer of stations 01, 18 and 27 indeed result from the presence of large PP particles with respective mean (± s.d.) Feret dimeters of 137±110 µm (n=22), 172±135µm (n=10), and 380±253 µm (n=3). Note that the contribution of PP microplastics with Feret diameter larger than the average size of all detected PP microplastics (116±35µm) was 39% at station 01, 60% at station 18, and 100% at station 27. Thus, adding a random large PP particle would thus not make a significant change to the mass concentrations and size structure of PP debris found in the mesopelagic layer.*

*The scarcity of PS microplastics in some of our samples, and specifically those collected in the deepest water layer and in the South Atlantic is discussed in lines 233-243. We agree with the reviewer that filtering more water and imaging larger areas on the filter might reduce the uncertainty of the measurements, especially for plastics which have relatively low abundance in the environment. In support of Lebreton et al. 2017, we also encourage the adoption of class-based (e.g. polymer type and size) methodologies for collection, extraction and analysis of plastic litter as a way to improve data quality in future studies (lines 244-254).*

DISCUSSION

**Comment 25:** Overall, the discussion is excessively long and too speculative with a highly variable dataset that doesn’t support most of the comments, or being so variable, it’s not even worth discussing. Drastically reduce this section to the robustly findings supported by the data. For example: all discussion about PS (its detection is too variable, no need to speculate).

***Response 25:*** *The discussion section has been revised following the recommendations of both reviewers.*

*Despite their high variability, our findings about PS concentrations in the Atlantic reveal several aspects about microplastic contamination which are important to discuss. To be more specific: while scarce and irregular presence of PS in surface waters was reported in earlier surveys, our study reveals low abundance of this polymer in the ocean interior, and especially in the South Atlantic. We also report a N-S decline in PS concentrations, which was not observed for either PE or PP microplastics. Our results demonstrate that different polymer types have different sources and fates in the ocean, and thus, it is important to classify plastic into classes (e.g. polymer types) and not regard it as a generic material, as suggested by Lebreton et al. 2017. This class-based view of plastic contamination is especially important to adopt if we are to improve sampling and detection methodologies, especially when quantifying the abundance of more ‘rare’ plastics, such as PS.*

**Comment 26:** Also reduce repetitiveness. For example: lines 150-161 overlaps with discussion in section line 188.

***Response 26:*** *The revised discussion section of the manuscript includes this suggestion of Reviewer #1.*

**Comment 27:** Line 171. EPS has so little density that it wouldn’t even enter the water. They are usually just blown to the coast.

***Response 27:*** *In the revised manuscript, we revisited the discussion of the factors that are likely to influence the abundance of PS microplastics in the ocean interior, and EPS grade of this material in particular (lines 234-243).*

**Comment 28:** Line 194. Microscopy or spectroscopy?

***Response 28:*** *FTIR imaging which is a combination of spectroscopy and imaging microscopy.*

**Comment 29:** Lines 188-214. While comparing their concentrations with other studies, authors need to discuss their results in terms of location. The most striking finding is that maximum densities are NOT found in the gyres, a well-established hotspot in previous studies.

***Response 29:*** *The spatial context of our findings was discussed in lines 180-194. The comparison of the basin-wide patterns of surface and subsurface abundance of microplastics was included to the revised discussion section (lines 180-214) and also in response to Comments 7 and 20.*

**Comment 30:** Lines 265-272. This back-of-the-envelope calculation, and which is the major conclusion of this study, need a bit more accurate estimation. Not taking the average of all vertical and horizontal distribution, but modelling these distributions and doing a more accurate calculation of the total budget.

***Response 30:*** *There is a temptation to assume that modelling leads to greater accuracy and more reliable conclusions. The authors are great enthusiasts of modelling when they can be parameterized with confidence. In the case of the work we report, a model could easily be constructed which encapsulates* *the processes which determine the distribution of plastics (source locations, supply terms, sinking rates, fragmentation rates etc). Such a model would give the impression that our understanding is much better than is actually is the case. We would challenge anyone to provide parameterisations which have confidence levels better than plus or minus an order of magnitude. Such model construction would be completely misleading and we do not think it is ethical to present such results. It is much more honest and clear to present very simple calculations which tell a very simple and powerful story.* *There is certainly spatial variability which we cannot at present explain with confidence but this in no way reduces the conclusion from the paper, one which radically alters our understanding of plastic pollution of the ocean.*

M&Ms

**Comment 31:** The methods description is very thorough and sometimes difficult to follow… From what I understand, SAPs collected the particles on 55 and 1 um filters that were then rinsed off with ASW. Were both filters mixed? Do authors have what was collected on the 55um and 1um filters separately?

***Response 31:*** *We apologise if the description of the methods was confusing for the reviewer. The processing of the ASW-particle mixture prior to FTIR imaging is now clarified in lines 415-431. To clarify here: post-rinsing with ASW, two individual size fractions were sequentially filtered into a 25 mm stainless steel disk with the mesh size of 25 µm.*

**Comment 32:** Lines 383-385. This data needs to be included to have a full picture of the total budget. Now I understand why the smallest particle found was of over 30um… why wasn’t the silver 0.8um filters analysed and reported here?

***Response 32:*** *As in our response to Comments 2 and 3 of Reviewer #1, we agree that quantifying microplastic of smaller size category would indisputably improve our understanding of the oceanic plastic burden. Here we report the size fraction of polymer-specific microplastics which is well above the detection limit as these data are of the highest confidence. We refer to the smaller size fraction of our samples (< 25 µm) to ensure that the procedure we followed could be reproduced in future studies. We would like to emphasize that our consistent measurements of small microplastics across the wide geographical region and at several discrete depths are the first of their kind in studies of marine plastic contamination. Focussing only on three polymers of > 25 µm size category distributed in the subsurface ocean, we were able to balance the budget of marine plastics and to demonstrate that the previous works were insufficient to capture the crucial size fraction of these contaminants. We also emphasize that accounting for microplastics <25 um in size, including nanoplastics, will only improve the representation of the sink term of the mass-balance. The inputs of plastic to the ocean, which are currently highly uncertain due to the lack of the robust empirical basis, must be constrained to the same degree (e.g. polymer type, particle size) as the sink terms to obtain the full picture of plastic budget in the ocean.*

**Comment 33:** Line 386. I am not aware of the exact methodology of FTIR imaging and, hence, can’t revise this section correctly. But it would be nice to have a better explanation of all the 6 x 6 mm2 and 3 x 3 mm2 and sections, etc. It’s a bit confusing what was imaged and how the data was extrapolated (line 425). Couldn’t the whole filter be imaged?

***Response 33:*** *The choice of the* *6×6 mm^2^ section for FTIR imaging was determined by the aperture of the sample holder (Ø=10 mm) on which the stainless-steel filter was mounted for transmission measurements. To prevent sample loss, potential contamination, and double-counting of particles, we did not reposition the filter on the sample holder in order to expose other areas for imaging (lines 444-448).*

**RESPONSE TO REVIEWER #2’S COMMENTS**

Reviewer #2 (Remarks to the Author):

**Comment 1:** 1. Comments for Author

In this paper Pabortsava1 & Lampitt conducted during the 26th ATM cruise microplastic (MP) sampling on a latitudinal transect across the Atlantic (~ 46°N – 53°S) in September to November 2016. They filtered large volumes of water at three-selected depth (surface, intermediate, mesopelagic) based on hydrographic settings. From the samples, small aliquots were taken and after removal of organic material, the filters were subjected to MP measurements using Fourier-Transform infrared (FTIR) imaging down to a resolution of 25 µm pixels. The study focussed on the three, commercial most prominent polymer types of polyethylene (PE), polypropylene (PP) and polystyrene (PS). Beside particle number they also provide the polymer-specific mass concentrations, which was calculated based on particle size and shape, including different test to have minimum and maximum values for this type of conversion.

The average observed polymer weight from the various ocean depth were further basin wide upscaled and discussed, how MP in the size range of 32-651 μm will contribute to the overall plastic pollution budgets, previously estimated. The authors claim that with their study they can show that there is not a missing sink for plastic in the ocean, but rather, even when focussing only on three compounds (PE, PP &PS) and the small particles, they can already account for all the cumulative plastic supply reaching the Atlantic Ocean since the early 50ties. Thus, they consider that the overall input of plastic litter into the ocean must be much larger then hitherto accounted for. However, this conclusion is based on a budget, which is not properly presented in the manuscript and will need a revision, before the paper can be accepted.

The role of plastic litter in the ocean is of major concern with high societal relevance. The authors present the first measurements focussing on small particles of 32-651 μm, on an ocean wide transect covering different geographic regions in oceanography and distinct depth horizons and thus shed new light on the overall plastic pollution in a global context. However, these are only 12 stations and the question remains, how representative these sample collections are for the chosen transect. Given the long history of AMT cruises, the authors should discuss this in a broader context.

Overall, sampling and measurements appear to be of high quality and the provided contamination measures seem to be appropriate for this type of analysis. The only unclear part of the investigation is the use of a Folsom splitter and the resulting effect on the overall numbers derived in this study. In addition, parts of the discussion are inconsistent and need some revision.

Overall, I assume that this study will rock the field of microplastic research, since it again puts the attention to the very small MP, which are not captured with common methods as manta trawls, which are usually recommended to use for monitoring of surface sea water (e.g. GESAMP (2019). Guidelines or the monitoring and assessment of plastic litter and microplastics in the ocean). This type of sampling devices only capture particles larger than 300µm, which according to this study are of minor importance in the overall context of plastic weight in the ocean. If they can come up with a convincing basin-scale budget, it will also stimulate the discussion about re-evaluation of plastic sources for the marine environment. Statistical analysis have been performed adequately and the overall detailed description of sample handling and identification procedure seem to be appropriate, thus other scientists in the field should be able to replicate this type of MP measurements.

Please find below more specific points, which need some attention before the publication can be accepted:

***Response 1:*** *We thank Reviewer #2 for the constructive and helpful feedback on our manuscript and supporting its publication in Nature Communications. We are especially pleased to see that Reviewer #2 thinks that our study* ***‘will rock the field of microplastic research’*** *and recognises that our sampling and measurements are of* ***‘high quality’****,* ***‘appropriate’*** *and that they can be replicated by other scientists.*

*The revised manuscript now includes the suggestions and recommendations of Reviewer #2. Specifically, we improved the presentation of oceanic plastic budget in the main article and provided the detailed description of calculations and data used in the Methods section and in Supplementary Information. We clarified the use of Folsom splitter on the reported concentrations of microplastics and improved the overall discussion of our observations by incorporating the feedback of both reviewers. We also discussed our sample collection in the context of the annually repeated Atlantic Meridional Transect programme. Below, we respond to specific comments and suggestions of Reviewer 2.*

Abstract:

**Comment 2:** Line 9: It need to be clarified, how the basin-scale numbers were obtained. (see again below)

***Response 2:*** *The methods section of the revised manuscript includes the detailed description of the basin-scale calculations of the plastic mass balance in the Atlantic Ocean (lines 511-543) with all relevant values also provided in the Supplementary Table S2.*

**Results**

**Comment 3:** Line 71: employing a fixed temperature-based criterion of ref24. Please explain in more detail which MLD criteria has been used.

***Response*** ***3:*** *The selection of isothermal layer marking the location of the base of mixed layer has been clarified in lines 69-76. Per suggestion of Reviewer #1, the revised Fig. 1b now refers to ‘depth’ instead of ‘pressure’*.

**Comment 4:** Line 75: also refer to Supplementary Table S1

***Response 4:*** *Done.*

**Comment 5:** in all procedural blank. Please explain better and refer to the details in the supplements.

***Response 5:*** *The contamination free sampling and analysis has been clarified in lines 77-86 with relevant references to supplementary figures and methods added.*

Discussion:

**Comment 6:** Overall, the discussion is not consistent as to why certain types of polymers float or sink and requires clear lines of argumentation in the revised form of the manuscript. In addition, the authors should put more attention to the fact, how reliable their basin-scale estimates are.

***Response 6:*** *The discussion section has been revised and now incorporates the suggestions made by both reviewers. Specifically, in lines 180-208 and 219-243 we revised the discussion about the factors that could influence the distribution of the examined polymer types. We clarified the calculation of the oceanic plastic budgets in the main text (lines 300-309) and included detailed description in the methods section (lines 511-543) and provided relevant data in the Supplementary Table S2. The appraisal of our mass-balance calculations was addressed in lines 290-299.*

**Comment 7:** Line 159ff: Please give references to this statement, what specific gear is used. Other studies in the Southern Ocean also rather find PA in their samples, which is usually attributed to fishing gear.

***Response 7:*** *The relevant references have been included into the revised version of the manuscript (lines 226-228). To be more specific here: two different longline methods have been consistently used in the South Georgia fishery: the traditional “Spanish” system and the autoline, also known as Mustad© system. The Spanish system is characterised by the deployment of two separate lines - main (braided polypropylene rope) and fishing lines (formed by a junction of line segments of either monofilament nylon or braided polypropylene) connected by the connecting line (see Fig. 2.5 in Benedet (2017), Kokorin and Istomin (2006) and Robertson et al. (2008)). The ‘bottom’ Spanish longline is with a hooking snood is made of weaved polyethylene strings. Vertical pole buoys are made of EVA or PE shell and EPS foam filling. The autoline system consists of a braided main line made from a combination of polypropylene and polyethene (see Fig. 2.7 in Benedet (2017) and references therein).*

*Pelagic (mid-water) trawls are used to catch Antarctic krill (Krag et al. 2014). While the nets of such trawls are often made of PA, the ropes are typically manufactured from either PE or a composite of PE and PA (see for example, DYNAMICA ropes (https://dynamica-ropes.com/)).*

**Comment 8:** Line 169 ff: Sentence seemed to be out of context and wrong since PE and PS are typical lighter compared to seawater, as the authors state in their own supplements Line 622 ff ((PE=0.880-0.960 g cm-3; PP=0.895-0.92 g cm-3; PS=1.04–1.05 g cm-3)

***Response 8:*** *We revised the section discussing the observed distribution of PE, PP and PS in our study region (lines 229-243).*

**Comment 9:** Line 181: “To address…..” Please specify in more detail

***Response 9:*** *We specified the way to reduce the uncertainties in quantifying polymer-specific microplastics in lines 247-254.*

**Comment 10:** Line 184-185: Please give reference

***Response 10:*** *The reference is now included line 253. Since the methods for sampling and analysis of major and trace metal constantly evolve, here we reference ‘Sampling and Sample-handling Protocols for GEOTRACES Version 3, 2017 (Cookbook, version 3.0, 2017; Cutter et al. 2017)’. This is a compilation of the most reliable protocols to date for sampling and analysis of dissolved and particulate elements (major, trace, isotopes) at sea edited by the 2017 GEOTRACES Standards and Intercalibration Committee.*

**Comment 11:** Line 202: Incorrect, numbers in sea ice were orders of magnitude higher.

***Response 11:*** *we thank the reviewer for spotting the error. In the revised manuscript we report microplastic abundance in sea ice (Peeken et al. 2018) in correct concentration units (lines 159-162).*

**Comment 12:** Line 206: How do data compare to the North Atlantic (e.g. Lusher et al. 2015)

***Response 12:*** *Comparison to the observation of Lusher et al. 2014 in the Northeast Atlantic were included in line 159.*

**Comment 13:** Line 226: “short residence time of…” This seems not so clear from the data presented in Fig 4, which show only for PP the short residence time in the surface, while PE and PS decrease with depth.

**Comment 14:** Line 227ff: Line of arguments thus not clear

***Response 13 and 14:*** *This section of the discussion was revised (lines 255-277).*

**Comment 15 and 16:** Line 260ff: How were the estimates done; maybe this section is missing in the result part? Line 266: What is the whole area?

***Response 15-16:*** *We clarified the plastic budget calculations in the main text (lines 300-309) and added a detailed description to the methods section (lines 511-543). We also provided the relevant reference data in Supplementary Table S2.*

**Comment 17:** Line 274: 6%, how is this number derived?

***Response 17:*** *We apologise for quoting a rounded value in the earlier version of the manuscript. The* *volume of the top 200 m of the Atlantic Ocean was calculated to be 5.3% of its total volume (3.1×10^8^ km; Eakins 2010). The latter was calculated by multiplying the area of the Atlantic Ocean (8.2 ×10^7^ km^2^; as the North and South basins combined and excluding the Mediterranean and the Baltic Seas; Eakins 2010) by the average depth of the North and South Atlantic basins (3746 m; Eakins 2010). Similar calculation was performed for the 200 m layer of the Atlantic.*

**Comment 18:** Line 302: Arctic not Antarctic

***Response 18:*** *Corrected.*

Methods:

**Comment 19:** Line 353: “thoroughly cleaned prior to every” How were they cleaned?

***Response 19:*** *The procedure for cleaning laboratory ware and filters is described in Supplementary methods (lines 611-633).*

**Comment 20:** Line 374ff: If you split 1000ml you should receive 250 ml. How was the next step of aliquots done, since only 100ml were used for further treatment of the samples?

***Response 20:*** *The procedure of splitting the particle-water mixture was clarified in lines 419-424 of the revised manuscript. To specify here, the glass flask containing the ASW-particle mixture was swirled to homogenise the sample and poured into another graduated glass flask (200 mL) up to a mark of 100 mL. Note that during sample handling we used as few containers as possible to minimise the sample contamination as well as particle loss.*

**Comment 21:** Which effect has the Folsom splitter on MP contamination and did you test, how this affects your overall calculation of concentrations?

***Response 21:*** *Folsom splitter is made of Polymethyl Methacrylate (Plexiglass/Perspex) wheel and cups and a wooden base. Plexiglass is a different polymer material to those reported in this study. This polymer was not identified in any of our sample blanks.*

Data availability:

**Comment 22:** Line 462: For publication in Nature Communications I think it is required to upload the data in a public data base.

***Response 22:*** *The microplastics data reported in this study will be uploaded to British Oceanographic Data Centre (BODC), a public data base, as required.*

**Comment 23:** In conclusion, I think this paper is highly valuable to be published in Nature Communication however; I think the authors need to clarify the budget section of the paper and fix a few more inconsistencies before the paper can finally be accepted.

***Response 23:*** *we thank the reviewer for the constructive feedback, very useful suggestions and overall very positive comments about our work.*

**References**

1. Dehaut, A., Cassone, A-L., Frère, L., Hermabessiere, L., Himber, C., Rinnert, E.*, et al.* Microplastics in seafood: benchmark protocol for their extraction and characterization. *Envir. Poll.* 2016, **215:** 223-233.
2. Kühn, S., van Werven, B., van Oyen, A., Meijboom, A., Bravo Rebolledo, E.L., van Franeker, J.A. The use of potassium hydroxide (KOH) solution as a suitable approach to isolate plastics ingested by marine organisms. *Mar. pollut. bull.* 2017, **115**(1)**:** 86-90.
3. Löder M.G.J., Gerdts G. (2015) Methodology Used for the Detection and Identification of Microplastics—A Critical Appraisal. In: Bergmann M., Gutow L., Klages M. (eds) Marine Anthropogenic Litter. Springer, Cham
4. Elert, A.M., et al. Comparison of different methods for MP detection: What can we learn from them, and why asking the right question before measurements matters? Environmental Pollution 231, 1256-1264 (2017).
5. Geyer, R., Jambeck, J.R., Law, K.L. Production, use, and fate of all plastics ever made. *Science advances* 2017, **3**(7)**:** e1700782.
6. Erni-Cassola, G., Zadjelovic, V., Gibson, M.I., Christie-Oleza, J.A. Distribution of plastic polymer types in the marine environment; A meta-analysis. *J. Hazard. Mater.* 369, 691-698 (2019).
7. Law, K.L., Morét-Ferguson, S., Maximenko, N.A., Proskurowski, G., Peacock, E.E., Hafner, J.*, et al.* Plastic Accumulation in the North Atlantic Subtropical Gyre. *Science* 2010, **329**(5996)**:** 1185.
8. Eriksen, M., Lebreton, L.C., Carson, H.S., Thiel, M., Moore, C.J., Borerro, J.C.*, et al.* Plastic pollution in the world's oceans: more than 5 trillion plastic pieces weighing over 250,000 tons afloat at sea. *PloS one* 2014, **9**(12)**:** e111913.
9. Cózar, A., Echevarría, F., González-Gordillo, J.I., Irigoien, X., Úbeda, B., Hernández-León, S.*, et al.* Plastic debris in the open ocean. *PNAS* 2014, **111**(28)**:** 10239-10244.
10. Enders, K., Lenz, R., Stedmon, C.A., Nielsen, T.G. Abundance, size and polymer composition of marine microplastics≥ 10 μm in the Atlantic Ocean and their modelled vertical distribution. *Mar. pollut. bull.* 2015, **100**(1)**:** 70-81.
11. Bergmann, M., Wirzberger, V., Krumpen, T., Lorenz, C., Primpke, S, Tekman, M.B.*, et al.* High quantities of microplastic in Arctic deep-sea sediments from the HAUSGARTEN observatory. *Environ. sci. technol.* 2017, **51**(19)**:** 11000-11010.
12. Kanhai, L.D., Officer, R., Lyashevska, O., Thompson, R.C., O'Connor, I. Microplastic abundance, distribution and composition along a latitudinal gradient in the Atlantic Ocean. *Mar. pollut. bull.* 2017, **115**(1-2)**:** 307-314.
13. Mitrano, D. (2019). "Nanoplastic should be better understood." Nature Nanotechnology 14(4): 299-299.
14. Booth, A.M., Kubowitcz, S.K., Beegle-Krause, C.J., Skancke, J., Nordam, T., Landsem, E., Throne-Holst, M., Jahren, S. Microplastic in global and Norwegian marine environments: Distributions, degradation, mechanisms and transport.). SINTEF Oceans AS (2017).
15. Wright, S.L., Kelly, F.J. Plastic and Human Health: A Micro Issue? *Environ. Sci. Technol*. 51, 6634-6647 (2017).
16. Lebreton, L.C., Van der Zwet J., Damsteeg J-W., Slat, B., Andrady, A., Reisser. J. River plastic emissions to the world’s oceans. *Nat. commun.* 2017, **8:** 15611
17. Benedet, R. A. (2017). The bottom longline fishery and its use as a source of benthic biodiversity information around South Georgia. Faculty of Science, Technology, Engineering and Mathematics (STEM). British Antarctic Survey, he Open University. PhD.
18. Kokorin, N. V., Istomin, I.G. (2006). "Use of a deep-water longline of the “Spanish type”and his modifications in the Russian research of Ross Sea toothfish during the season 2004/05-2005/06." CCAMLR WG-FSA-06/5.
19. Robertson, G., Moreno, C.A., Gutiérrez, E., Candy, S.G., Melvin, E.F., & Seco, Pon, P.S. (2008). "Line weights of constant mass (and sink rates) for Spanish-system Patagonian toothfish longline vessels." CCAMLR Science(15): 93-106.
20. Krag, L. A., et al. (2014). "Size Selection of Antarctic Krill (Euphausia superba) in Trawls." PloS one 9(8): e102168.
21. Cutter, G., Casciotti, K., Croot, P., Geibert, W., Heimbürger, L-E, Lohan, M., Planquette, H., van de Flierdt, T. Sampling and Sample-handling Protocols for GEOTRACES Cruises. In: Cruise and Methods Manual (CookBook)). GEOTRACES International Project Office (2017).
22. Peeken, I., Primpke, S., Beyer, B., Gütermann, J., Katlein, C., Krumpen, T.*, et al.* Arctic sea ice is an important temporal sink and means of transport for microplastic. *Nat. commun.* 2018, **9**(1)**:** 1505
23. Lusher, A.L., Burke, A., O’Connor, I., Officer, R. Microplastic pollution in the Northeast Atlantic Ocean: Validated and opportunistic sampling. *Mar. pollut. bull.* 2014, **88**: 325-333.

Eakins, B. W. a. G. F. S. (2010). Volumes of the World's Oceans from ETOPO1. N. N. G. D. Center, NOAA National Geophysical Data Center

**Reviewer comments, second version:**

REVIEWERS' COMMENTS:

Reviewer #1 (Remarks to the Author):

The authors have done an excellent job in addressing most comments. The manuscript reads much better, and now data and methods are much clearer and better presented.

While I understand the statistical analysis of other materials is problematic due to their very low abundance during this survey, its availability would be of interest and uttermost importance to many readers. This data exists as authors refer to it in line 107. Hence, as also requested by the other reviewer, before this paper can be accepted for publication all data should be provided as a raw supplementary table with all fragments and material types (including PA, etc) found in all locations.

Other minor comments:

Authors correctly argue that modelling the distribution and total plastic budget would be incorrect (Response 30), but then mention that they “modelled estimates of plastic pollution in the Atlantic” (line 57). Readers will expect to see a model for the calculation. Maybe just saying “and estimate the plastic pollution…” would be enough to not raise reader’s expectations.

Line 175: Arctic

Line 318. I’m not sure if it’s worth talking about nanoparticles when the most abundant particles found in this study were not for the smaller fraction (25um) and, if I understand figure 3 correctly, smaller particle abundance seems to severely drop off with size. Hence, data suggests plastic particle number doesn’t follow a logarithmic increase expected from larger plastic fragmentation. This is actually an interesting observation previously seen in Cozar et al 2014 PNAS but in the mm range and surface seawater.

Reviewer #2 (Remarks to the Author):

The authors have considered my comments to my complete satisfaction
I thus recomend to accept the manuscript.

**Author rebuttal, second version:**

**GENERAL RESPONSE**

We are delighted that the editor and all the reviewers found our manuscript now entitled “**High concentrations of plastic hidden beneath the surface of the Atlantic Ocean**” suitable for publication in *Nature Communications*. We appreciate the time and efforts of the reviewers and editors in providing constructive comments and insightful suggestions. We have addressed all the issues indicated by Reviewers #1 and implemented the editorial suggestions. We believe that this revised version meets the publication requirements.

**RESPONSE TO REVIEWER #1’S COMMENTS**

Reviewer #1 (Remarks to the Author):

REVIEWERS' COMMENTS:

Reviewer #1 (Remarks to the Author):

**Comment 1:** The authors have done an excellent job in addressing most comments. The manuscript reads much better, and now data and methods are much clearer and better presented.

***Response 1:*** *We thank Reviewer #1 for another thorough and constructive feedback on our manuscript and for supporting its publication in Nature Communications. We have addressed all the issues and implemented the suggestions indicated by Reviewer #1. We believe that this revised version meets the publication requirements.*

*We respond to Reviewer #1’s individual comments below:*

**Comment 2:** While I understand the statistical analysis of other materials is problematic due to their very low abundance during this survey, its availability would be of interest and uttermost importance to many readers. This data exists as authors refer to it in line 107. Hence, as also requested by the other reviewer, before this paper can be accepted for publication all data should be provided as a raw supplementary table with all fragments and material types (including PA, etc) found in all locations.

***Response 2:*** *We provided the raw data table with all material (polymer) types found in our samples (as Supplementary Data 2) and their total number counts per area imaged. The spectral quality of the polymer types identified was also provided. We note that certain polymer types, such as rayon, cellulose/cotton, cellophane, as well as synthetic additives (plasticizers, lubricants, flame retardants, fillers, pigments) and coatings are listed on presence/absence basis. Rayon, cellulose/cotton and cellophane (regenerated cellulose) have similar spectra, which overlay each other on the IR images, making it impossible to reliably distinguish the substances (Comnea-Stancu et al. 2017, Primpke et al. 2018, Peeken et al. 2017). Their shape (fibrous) is also not suitable for FTIR imaging as the fibres do not lay flat on the filter surface, making them out of the focus of IR beam and increasing scattering (see for example, Peeken et al. 2017). Similarly, additives are structural components of other polymers and in some cases their spectra were interfering with other polymer types resulting in overlay on the IR image (see Fig. 2 in Primpke et al. 2018. Note that the spectral quality of additives in a measured spectral range was below the acceptance threshold.*

Other minor comments:

**Comment 3:** Authors correctly argue that modelling the distribution and total plastic budget would be incorrect (Response 30), but then mention that they “modelled estimates of plastic pollution in the Atlantic” (line 57). Readers will expect to see a model for the calculation. Maybe just saying “and estimate the plastic pollution…” would be enough to not raise reader’s expectations.

***Response 3:*** *This has been corrected per Reviewer’s suggestion.*

**Comment 4:** Line 175: Arctic

***Response 4****: Corrected accordingly. We thank the reviewer for spotting this.*

**Comment 5:** Line 318. I’m not sure if it’s worth talking about nanoparticles when the most abundant particles found in this study were not for the smaller fraction (25um) and, if I understand figure 3 correctly, smaller particle abundance seems to severely drop off with size. Hence, data suggests plastic particle number doesn’t follow a logarithmic increase expected from larger plastic fragmentation. This is actually an interesting observation previously seen in Cozar et al 2014 PNAS but in the mm range and surface seawater.

***Response 5:*** *We think it is important to refer to nanoplastics in line 322 as they are below the detection limit of the analytical method used in our study. Although Fig. 3 shows the decrease in particle abundance with size, targeted measurements of smaller plastic particles (<25 µm and nanoplastics) are required to confirm whether the abundance of the smaller plastic particles follow a logarithmic increase expected from larger plastic fragmentation.*

Reviewer #2 (Remarks to the Author):

**Comment 1:** The authors have considered my comments to my complete satisfaction. I thus recommend to accept the manuscript.

***Response 1:*** *We are delighted that Reviewer #2 supports the publication of our manuscript in Nature Communications and are thankful for the useful suggestions that have certainly improved the quality of our article*

**Reviewer comments, third version:**

**Author rebuttal, third version:**
